# Compact metamaterial-based single/double-negative/near-zero index resonator for 5G sub-6 GHz wireless applications

**DOI:** 10.1038/s41598-024-63610-x

**Published:** 2024-06-04

**Authors:** Sura Khalil Ibrahim, Samir Salem Al-Bawri, Mandeep Jit Singh, Husam Hamid Ibrahim, Mohammad Tariqul Islam, Md Shabiul Islam, Wazie M. Abdulkawi, Abdel-Fattah A. Sheta

**Affiliations:** 1 Department of Electrical, Electronic and Systems Engineering, Faculty of Engineering and Built Environment, Universiti Kebangsaan Malaysia, UKM, Bangi, 43600 Selangor, Malaysia; 2 Space Science Center, Institute of Climate Change, Universiti Kebangsaan Malaysia (UKM), Bangi, 43600 Selangor, Malaysia; 3 Faculty of Engineering, Multimedia University, Persiaran Multimedia, Cyberjaya, 63100 Selangor, Malaysia; 4 Department of Electrical Engineering, College of Engineering in Wadi Addawasir, Prince Sattam Bin Abdulaziz University, Al-Kharj, Saudi Arabia; 5 Electrical Engineering Department, King Saud University, 11421 Riyadh, Saudi Arabia

**Keywords:** DNG (double negative) metamaterial, ENG (electrical or epsilon negative), MNG (magnetic negative), Near-zero Index, SNG (single negative), Wireless communication, 5G sub-6 GHz, Electrical and electronic engineering, Materials for devices

## Abstract

The concept, performance, and analyses of distinctive, miniaturized metamaterial (MTM) unit cell addressing the forthcoming Sub 6 GHz 5G applications are presented in this paper. Two circular split-ring resonators (CSRR) with two parallel rectangular copper elements in front of the design and a slotted square element in the background make up the suggested metamaterial. It has a line segment with tunable features that is positioned in the center of the little ring copper structure. The suggested design offers a significant operating frequency band of 220 MHz together with a resonance of transmission coefficient S21 at 3.5 GHz. Furthermore, in two (z & x) principal axes of wave propagation, wide-range achievement, single/double-negative (S/DNG) refractive index, negative permittivity, and near-zero permeability properties were demonstrated. Through varying central slotted-strip line length, resonance frequencies can be selectively altered. Moreover, the metamaterial has overall dimensions of 9 × 9 mm^2^ and is composed on a Rogers 5880 RT substrate. In order to create the suggested MTM's equivalent circuit, which shows similar coefficient of transmission (S21), a proposed design’s numerical simulation is carried out in the CST micro-wave studio. This simulation is after that put to comparison with manufacturing of the design.

## Introduction

In the case when an electromagnetic wave interacts with a metamaterial, an artificially designed medium, it expresses extraordinary electromagnetic as well as optical properties. For a variety of uses in microwave communications, scientists from all around the world have focused on achieving such remarkable metamaterial features, like negative permittivity, permeability, and refractive index^1,2^. The applications encompass various fields such as improving the characteristics regarding antenna systems such as massive MIMO and MIMO^3–5^, designing absorbers^6^ sensors^7^, high-frequency communications^8^, optical communication^9^, harvesting energy^10^, remote aircraft^11^, microwave imaging^12^, metamaterial absorber^13^, and microwave devices like Bluetooth, WiMAX, GPS5^14^.

If the magnetic permeability (µ) or permittivity (ε) of the MTM is negative, it is referred to as SNG MTM. The epsilon negative (ENG) MTM is SNG MTM with negative value of ε, and the mu-negative (MNG) MTM is SNG MTM with negative value of µ. Finally, an MTM with a negative level of permittivity and permeability has been referred to as a DNG/LH MTM^15,16^. A metamaterial's unique and desired properties are determined by its shape, size, or geometry. Because matter has gravitational properties, light could be controlled by metamaterials, sound, EM (i.e., electro-magnetic) waves, and even electrical and mechanical forces^17–19^.

With respect to permittivity, permeability, and refractive index, they are specialized enough to have negative values. They are able to regulate or alter the material's permeability and permittivity in order to get the right behavior for some given use. They’re employed in order to enhance the functionality of couplers, antennas, and filters^20–22^.

Amazing concepts have been proposed by researchers to create a metamaterial with high effectiveness that is low-profile and economical. A metamaterial having double and single negative characteristics that is intended to function in microwave frequency bands below 6 GHz has been successfully constructed and analyzed. For the Koch Fractal MIMO Antenna Design for Sub-6 GHz V2X Communication at 5.9-GHz, a Double Negative (DNG) Metamaterial with 2 new, differently shaped left-handed meta-materials depending on broadside as well as electrically coupled square split-ring resonator (SRR) with negative values for each of permeability and permittivity have been proposed, and their reflection characteristics have been analyzed^23^. This study proposes a new asymmetric single split resonator metamaterial with negative permeability. The construction is a modified split ring resonator with a high EMR and a resonant frequency and bandwidth tuning mechanism^24^. For the purpose of forming MTS, placed on top of antenna (planer meta-surface (MTS) antenna), a 2 × 2 elliptical slot metamaterial unit cell is placed on top of Rogers RT-5880 substrate material with a dielectric constant of 2.2 and 0.0009^25,26^. Because of the decreased reflection coefficient caused by MTS use, the antenna's bandwidth appears at 520 MHz and 470 MHz (− 10 dB) from 2.57–3.09 GHz (520 MHz) and 3.61–4.08 GHz frequency, respectively. Considerable efficiency gains have also been seen, along with a noticeable 2–3 dB gain enhancement in the level of operating frequency. A tunable zero-order resonance (ZOR) active metamaterial antenna is suggested in^27,28^. It is employing a mushroom construction to achieve the narrow band. Since varactor diodes are specifically used in every unit cell, varying the voltage applied to them can alter their capacitance, which can be used to create tunable ZORs.

Because of its good narrowband properties in the Sub-6 GHz band, the suggested antenna could be deployed in the near future in 5G narrowband Internet of Things. The circular-shaped split-ring resonators (CSRR) which exhibit resonances of S21 covering 5G sub-6 gigahertz bands are given in this paper. They have two parallel rectangular copper in front of the design and a slotted square-shaped background tuned metamaterial. The design is unusual in that the length regarding slotted-strip line at the center of MTM's resonator patch could be adjusted to perform tuning properties. Additionally, at 3.5 GHz, this MTM offers negative permittivity, almost zero permeability, and negative refractive index. It also has negative permittivity, permeability, and refractive index, operating on X-axis. The remaining sections of the manuscript are arranged in the following way: "Unit cell meta-material design and simulation" will cover stages that are involved in design evaluation and the corresponding design and simulation of the circuit. The tuning attribute is covered in the second part, "Frequency tuning of the suggested MTM." The study of metamaterial performance and properties, like permeability, permittivity, impedance, and refractive index, also the examination of surface current, magnetic field, and electric field are covered in "Result, Analysis, and fabrication".

## Design of metamaterial unit cell (MTM) and simulation

### Single negative MTM (SNG)

**Figure 1:**
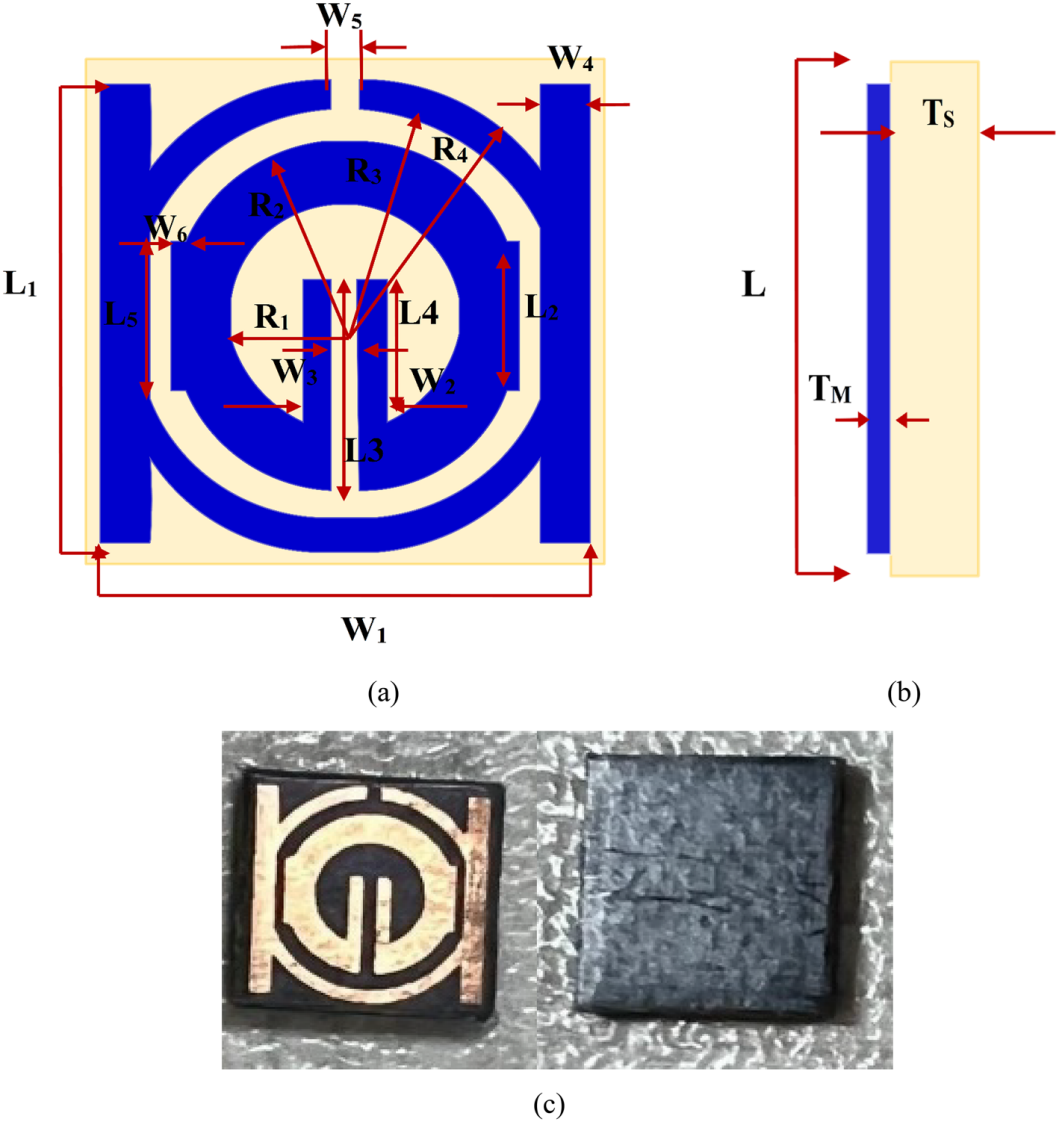
Proposed SNG-MTM unit cell: (**a**) simulated front view; (**b**) simulated side view; (**c**) Top and bottom of fabricated MTM.

The Rogers (RT-5880) substrate, which has a value of the dielectric constant ε_r_ of 2.2, a thickness of 1.575 mm, and a loss tangent δ of 0.0009, is the design basis for the suggested SNG-MTM unit cell. The chosen substrate dimension is 9 × 9 mm^2^. Copper (Cu), a conductor with a thickness of 0.035 mm with resistivity and conductivity values of 1.68 × 10^−8^ Ω.m and 5.8 × 10^7^ S/m, respectively, is used to form the resonating patch on this substrate material. Four circular-shaped CSRRs make up the unit cell. Two of them have a center split section on their upper arms, while the other two are divided on the corners of their right or left SSRR arms. For the purpose of changing the resonance frequency, the length of such central slotted-strip line is essential. This line's length can be adjusted to alter the resonances' frequency. The front view of the suggested unit cell's structural arrangement is shown in Fig. 1a, and the side view is shown in Fig. 1b. Table 1, shows all of the metamaterial parameters. The transmission capacity, coupling, and reflectance of the suggested and altered metamaterial shape could change. Figure 1 depicts the simulated and fabricated of metamaterial's overall unit cell configuration.

**Table 1:** Design parameters of proposed SNG-MTM unit cell.

Parameter	Dimensions (mm)	Parameter	Dimensions (mm)
L	9	W4	0.8
L1	8	W5	0.5
L2	2.6	W6	0.28
L3	3.79	R1	2
L4	2.55	R2	3.08
L5	2.92	R3	3.68
W1	8.4	R4	4.18
W2	1.5	T_S_	1.575
W3	0.5	T_M_	0.035

### Double negative MTM (DNG)

**Figure 2:**
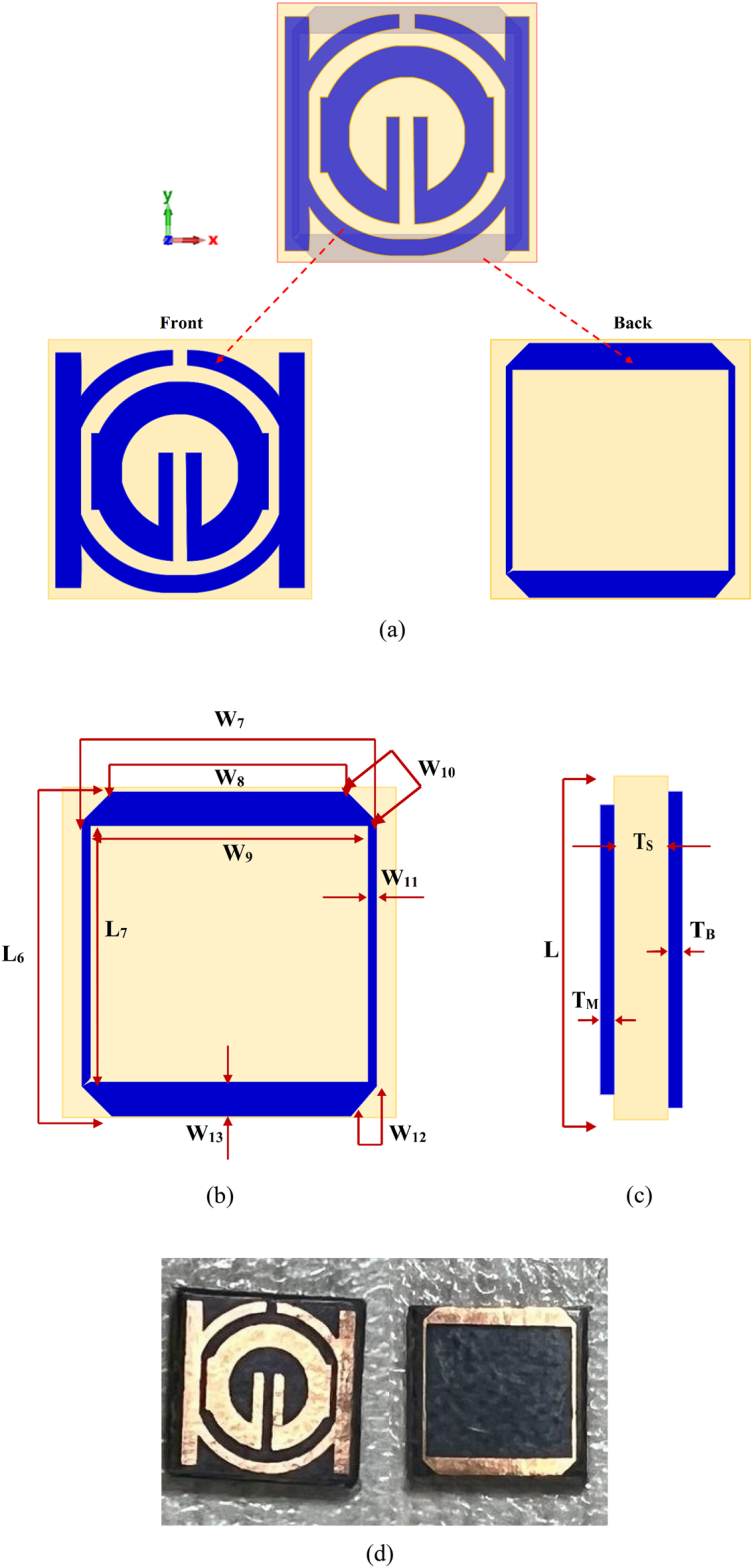
Proposed DNG-MTM unit cell: (**a**) simulated total, front and back view; (**b**) back side view; and (**c**) side view (simulated); (**d**) Top and bottom of fabricated MTM.

DNG-MTM unit cell consists of a square-shaped copper conductor that is positioned on the rear side of the design and two low profile complementary split ring resonators (CSRR) printed on the top layer with gaps comparable to SNG-MTM in section (A). The copper conductor with modified SNG-MTM properties is situated on the back side. Figures 2a–c show the dimensions regarding the side and back views of the design, respectively, and the total, front, and back views of the proposed unit cell. Table 2 displays metamaterial parameters. The transmission capacity, coupling, and reflectance of the suggested and altered metamaterial shape could change. Figure 2 depicts the simulated and fabricated of metamaterial's overall unit cell configuration.

**Table 2:** Design parameters of proposed DNG-MTM unit cell.

Parameter	Dimensions (mm)	Parameter	Dimensions (mm)
L6	8.8	W10	1.13
L7	7.2	W11	0.2
W7	8	W12	0.8
W8	6.4	W13	0.9
W9	7.6	T_B_	0.035

## Evolution steps of proposed unit cell

**Figure 3:**
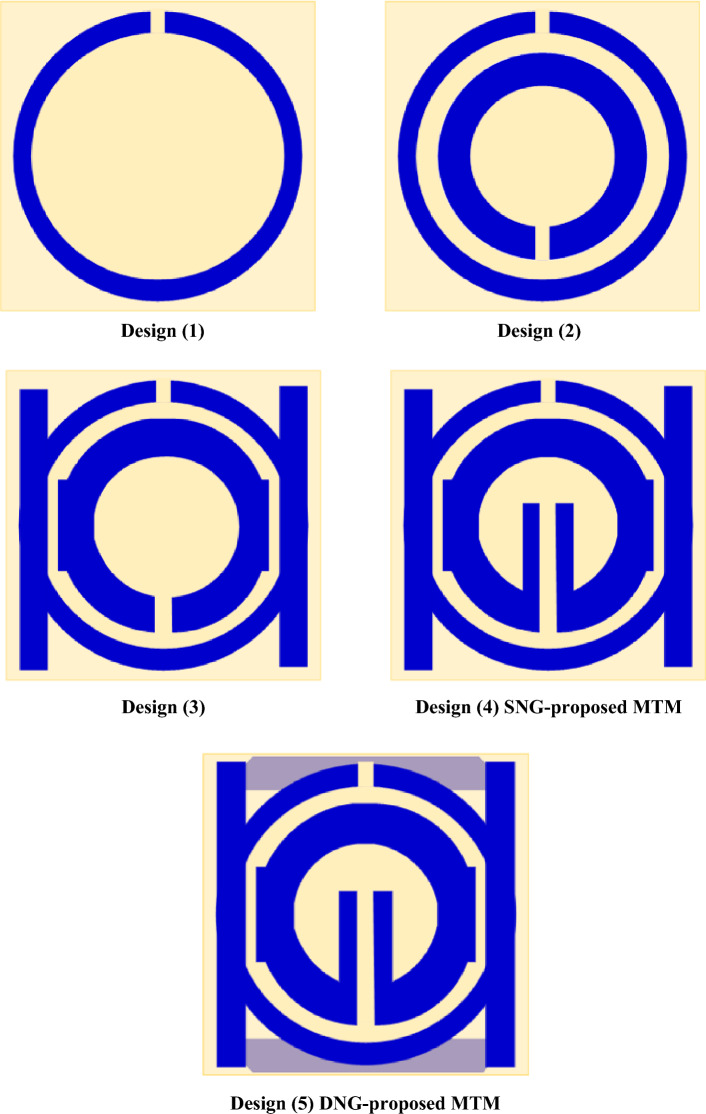
Evaluation steps toward the metamaterial design.

The suggested design of the MTM is finished step-by-step, as shown in Figs. 1 and 2, and through the monitoring of transmission coefficient's (S21) response for different configurations of the design, as shown in Fig. 3.

**Figure 4:**
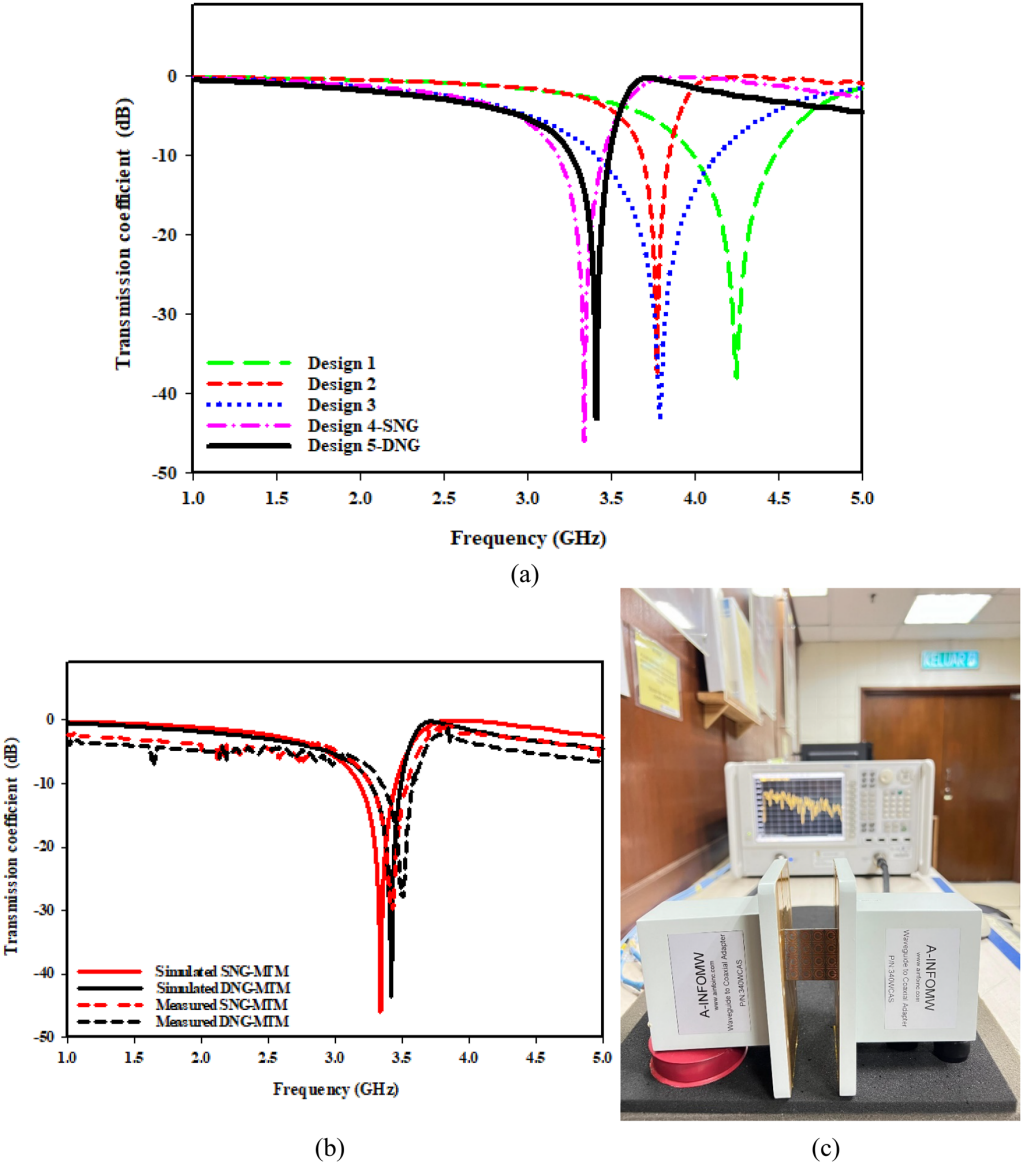
(**a**) Transmission coefficient for evolution steps toward the proposed MTM; (**b**) Simulated and fabricated of transmission coefficient with; (**c**) Setup for testing of proposed MTM.

Design (1) begins with circular ring that has a slot in the middle of the top, as shown in Fig. 3. At 3.5 GHz, this design exhibits a single resonance frequency. As seen in design (2), a slot is curved in the bottom middle and an internal ring, which is smaller than the outer ring, is added. 3.77 GHz is the resonance frequency provided by this configuration. Design (3) introduces a modest shift in the resonance frequency at 3.78 GHz by adding two parallel rectangular-shaped patches with varied sizes on two sides to the outer and inner rings. A shift in early resonance to 3.34 GHz occurs when a patch is introduced and slotted throughout its length with the bottom middle slot for the inner ring, as shown in design (4), due to the mutual inductance between the two- rings. Because of capacitive impact that the slotted-strip line produces, the negative MTM is formed by extending it until it reaches the inner ring. It is observed that the slotted-strip line is responsible for frequency shifting based on the resonant frequency movement capacity. To create a double negative MTM at resonance frequency 3.5 GHz, design (5) adds a square-shaped copper conductor to the back of the design. The coefficient of transmission for the steps of evolution toward the suggested MTM is shown in Fig. 4a,b, shows the simulated and measured transmission coefficient for the types of proposed designs whereas measurement setup is demonstrated in Fig. 4c. The peak value, ranges, and resonance frequency of the S-parameter are summarized in Table 3.

**Table 3:** The transmission coefficient (S21) of sequential unit cell steps.

Substructure	Resonance Frequency (GHz)	Bandwidth (GHz) at 10 dB	Resonance Peak (dB)
Design 1	4.24	4˗4.468	− 38.30
Design 2	3.77	3.682˗3.843	− 37.42
Design 3	3.78	3.422˗4.135	− 43.14
Design 4	3.34	3.172˗3.442	− 45.43
Design 5	3.5	3.30˗3.52	− 43.24

## Equivalent circuit modeling and simulation

**Figure 5:**
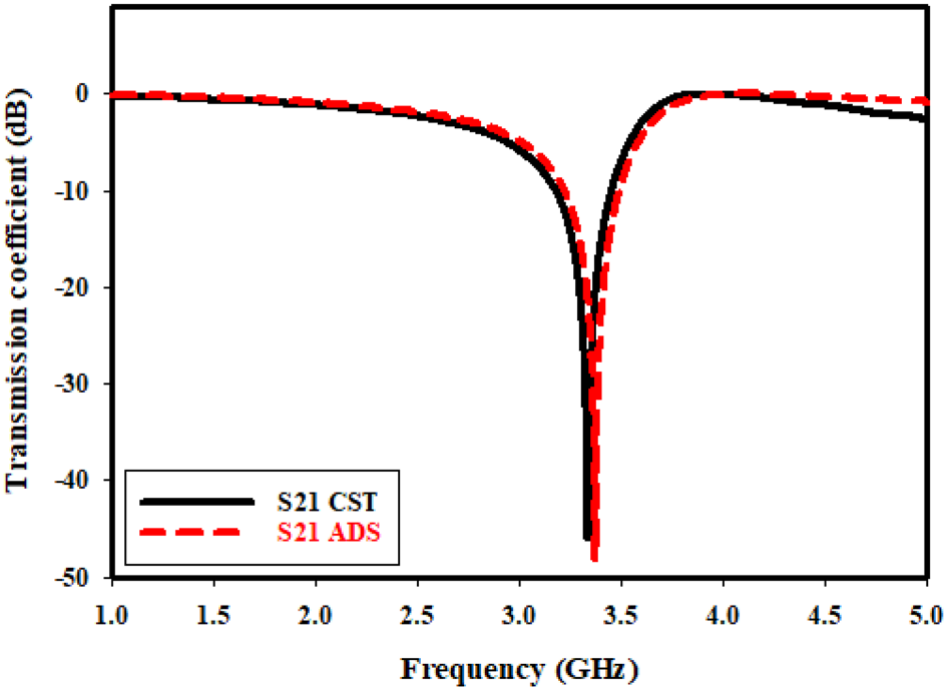
S21 comparison of the equivalent circuit result with simulation.

Researchers have tried their hardest to model the equivalent circuit multiple times. Through taking into consideration metallic conductors with inductor qualities, equivalent circuit of suggested meta-material unit cell can be constructed because of magnetic induction that is brought about by current flow. In order to ascertain whether similar circuit accurately depicts the intended unit cell, Fig. 5 contrasts the simulation outcomes of the CST and the advanced design system (ADS). It is clear that, despite having a smaller bandwidth and higher peak resonance, ADS and CST simulations yield results that are nearly comparable. The following formula^29^ can be used to get the metamaterial unit cell's resonance frequency (f_o_).

1 $${f}_{o}= \frac{1}{2\pi \sqrt{LC}}$$

Here, C and L stand for the capacitance and inductance of unit cell, respectively. Capacitance that is produced by gaps in rings and gaps between rings are expressed by the following equation:

2 $$C= {\varepsilon }_{o} {\varepsilon }_{r} \frac{A}{d}(F)$$

In which *ε*_*o*_ stands for the permittivity in free space and *ε*_*r*_ denotes relative permittivity. The metal strip or rings' break or gap's area is represented by the letter *A*, while its distance is indicated by the letter *d*. Depending on the transmission line concept, the next equation can be used to compute the metal strip's inductance^30^.

3 $$L=2\times {10}^{-4}l \left[\text{ln}\left(\frac{l}{w\times t}\right)+1.193+0.2235 \left(\frac{w\times t}{t}\right)\right]{k}_{g}$$

In which w stands for micro-strip line width, *l* for micro-strip line length, and *t* for micro-strip line thickness. *L* denotes the inductance in this equation and:

4 $$k_{g} = \left( {0.57 - 0.145 \ln \frac{{w^{\prime } }}{{h^{\prime } }}} \right)$$

Here $w^{\prime }$ is the width and $h^{\prime }$ is the thickness of the substrate^31^.

**Figure 6:**
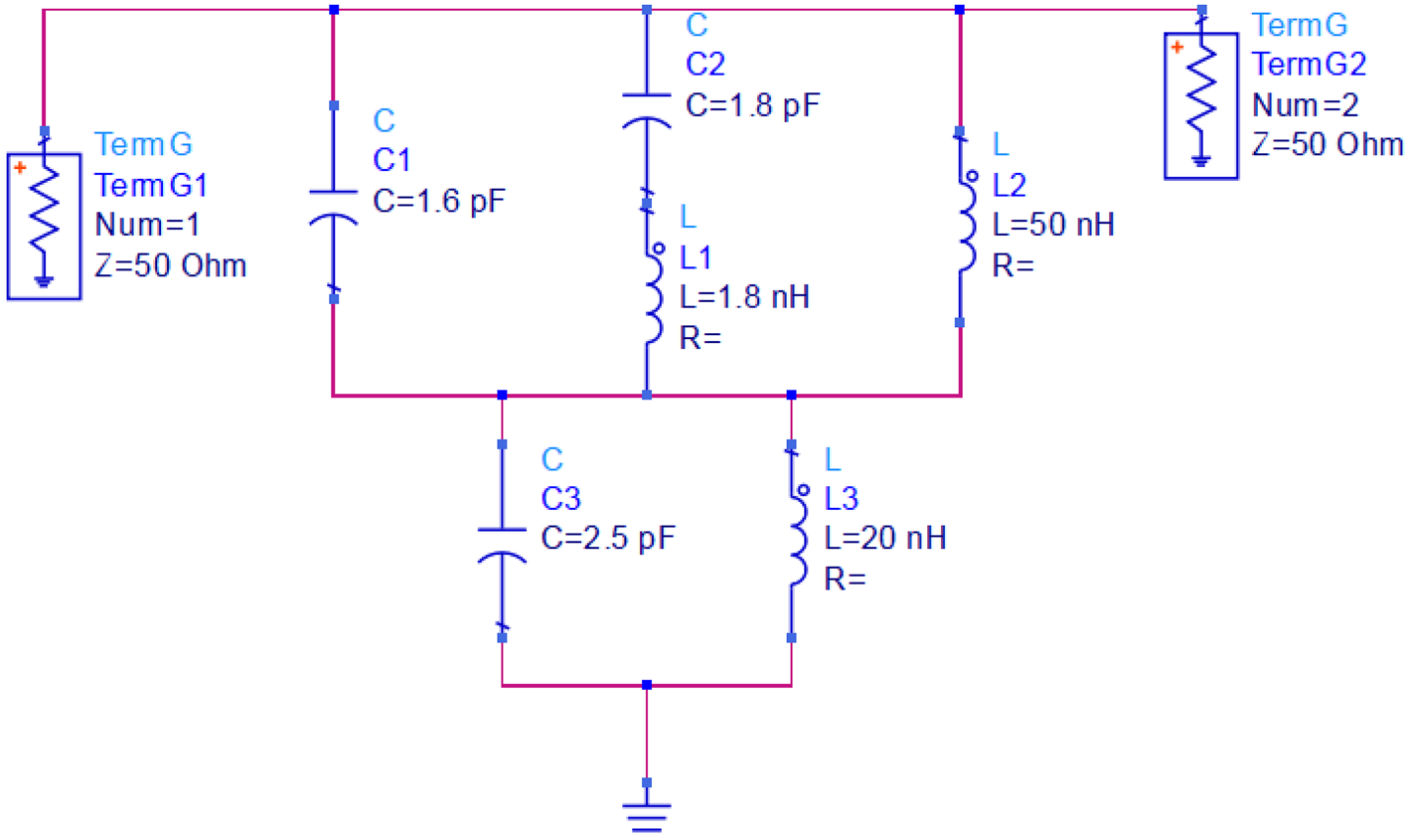
Equivalent circuit model.

It should be noted that MTM unit cell that is recommended contains both capacitive and inductive components, even though Fig. 6 depicts the evolution regarding equivalent circuit of suggested unit cell. Furthermore, as seen in Fig. 6, conducting strips of ring resonators form inductor (L) and gap or split in the ring, and area between rings is what makes up capacitor (C). Which is why, LC resonance circuit has been created by that MTM, illustrating resonances. For the purpose of the circuit analysis, the capacitor that has been created by split gap has been denoted by C1, C2, and C3, whereas patch lines have been represented as multiple inductors, L2, L1, and L3.

## Frequency tuning of the suggested MTM

**Figure 7:**
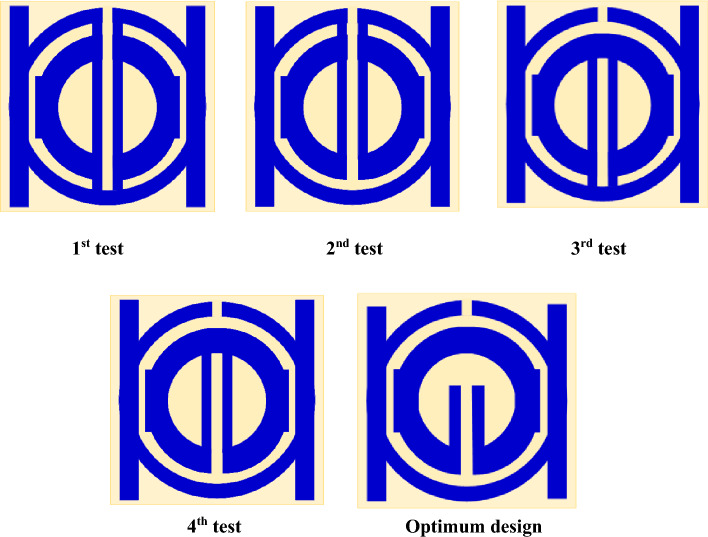
Tuning slotted-strip line of the proposed MTM.

The scattering qualities of the suggested MTM will be adjusted in accordance with application requirements by utilizing vertical strip line with a slot that is positioned in the middle, which has been shown in Fig. 7.

**Figure 8:**
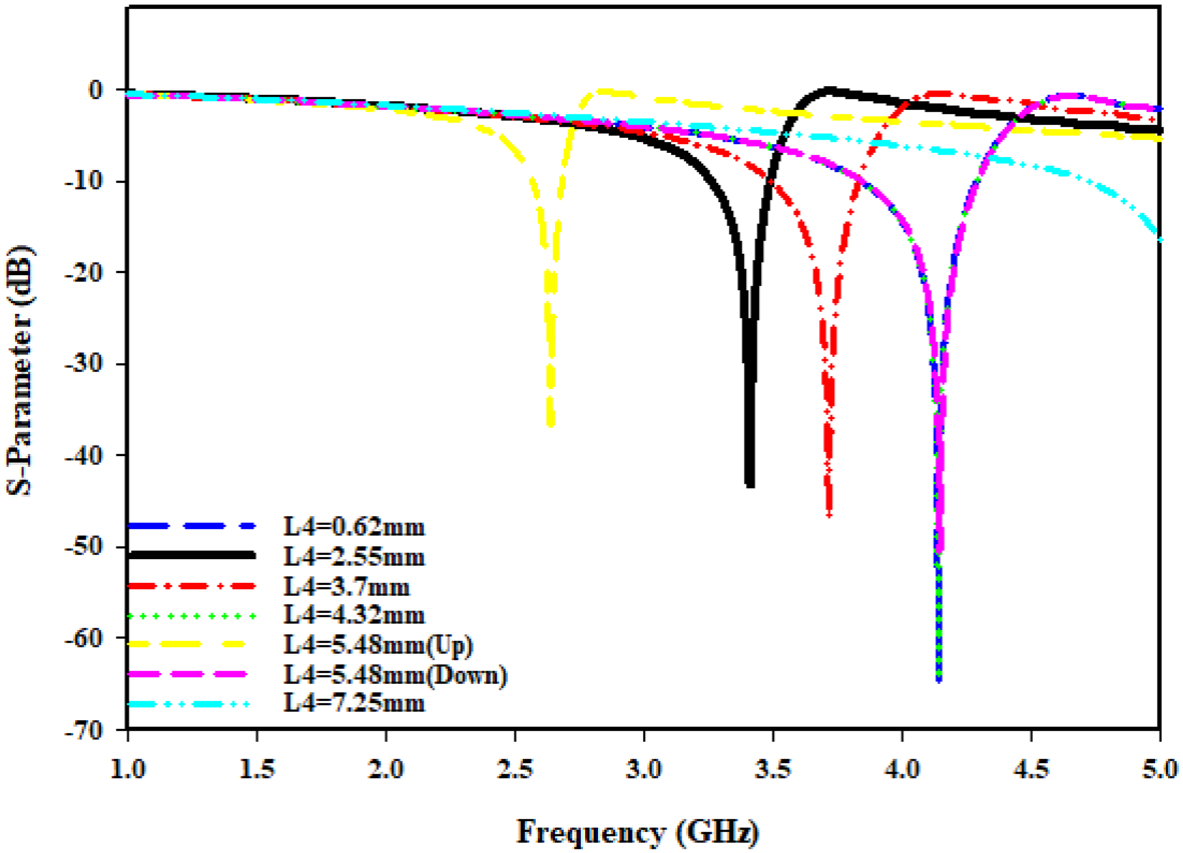
Transmission coefficient for evolution steps toward the proposed MTM with L4.

**Figure 9:**
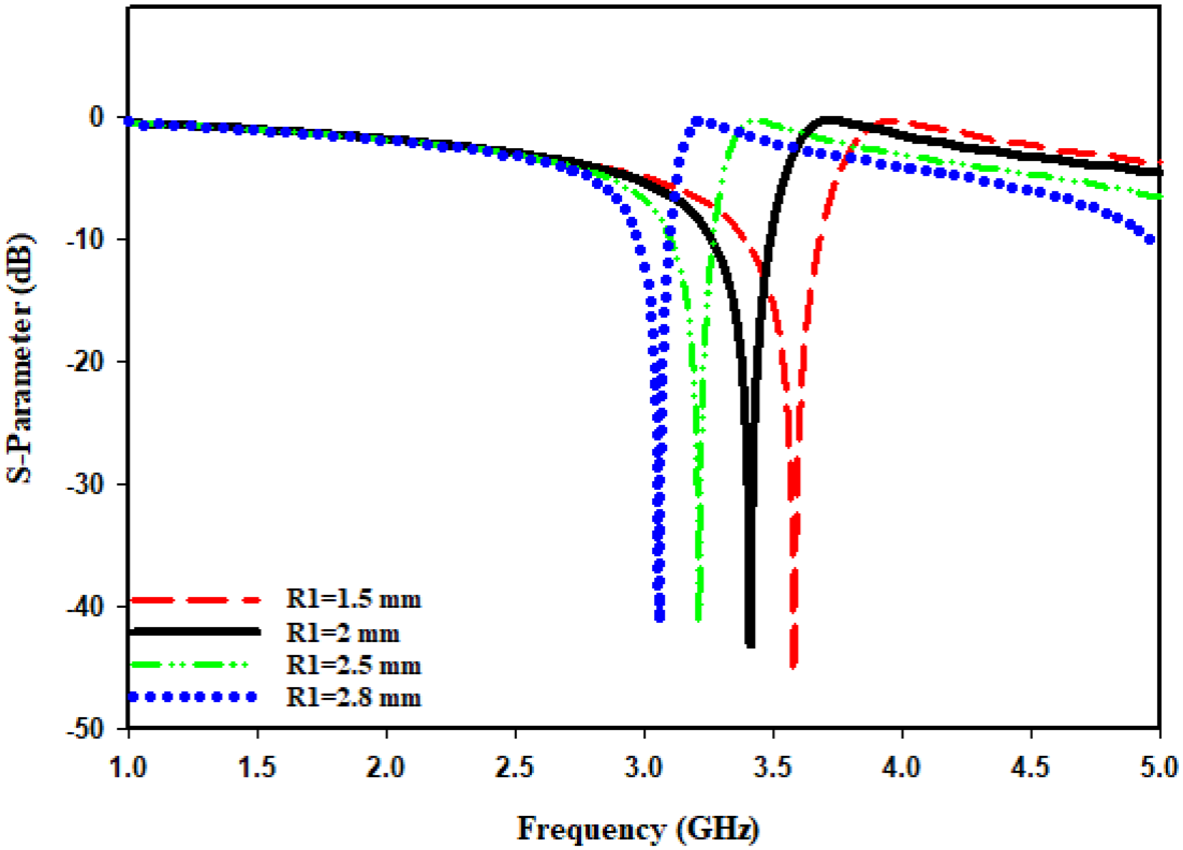
Transmission coefficient for evolution steps toward the proposed MTM with R1.

**Figure 10:**
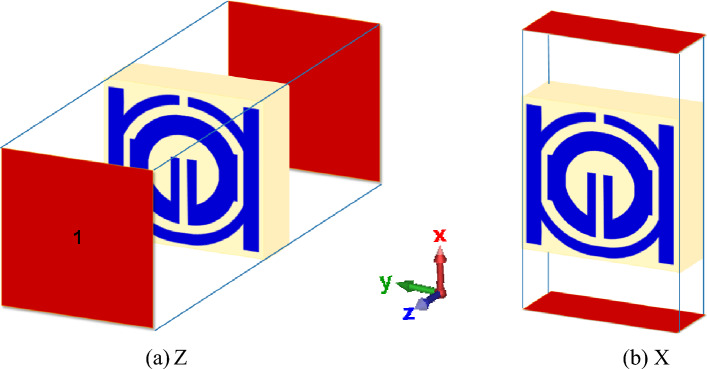
Metamaterial simulation set up: (**a**) on Z-axis; (**b**) on X-axis.

The radius (R1) from the center of the MTM to the inner ring is changed concurrently from minimum radius of 1.5 mm to maximum radius of 2.8 mm in order to maintain the monitor of the MTM's response. The length (L4) regarding such slotted-strip line is changed from minimum length of 0.62 mm to maximal length of 7.25 mm with a 0.5 mm wide slot. Resonances in the coefficients of transmission that are brought about by those modifications in the length of the strip line and radius R1 are shown in Figs. 8 and 9. The lowest bandwidth is 0.125 GHz with resonance at 3.058 GHz, while the largest bandwidth from variable radius is 0.292 GHz with a central frequency of 3.592 GHz. The lowest bandwidth is 0.124 GHz with resonance at 2.637 GHz, specifying that plane wave travels in Z-direction as well as electric and magnetic fields orient in X-direction and Y-direction, respectively, which has been illustrated in Fig. 10a. Maximum bandwidth from varying length is 0.460 GHz with a center frequency of 4.14 GHz. The effects of varying the radius and slotted split line on frequency and bandwidth as wave travels down Z-axis are compiled in Table 3.

**Figure 11:**
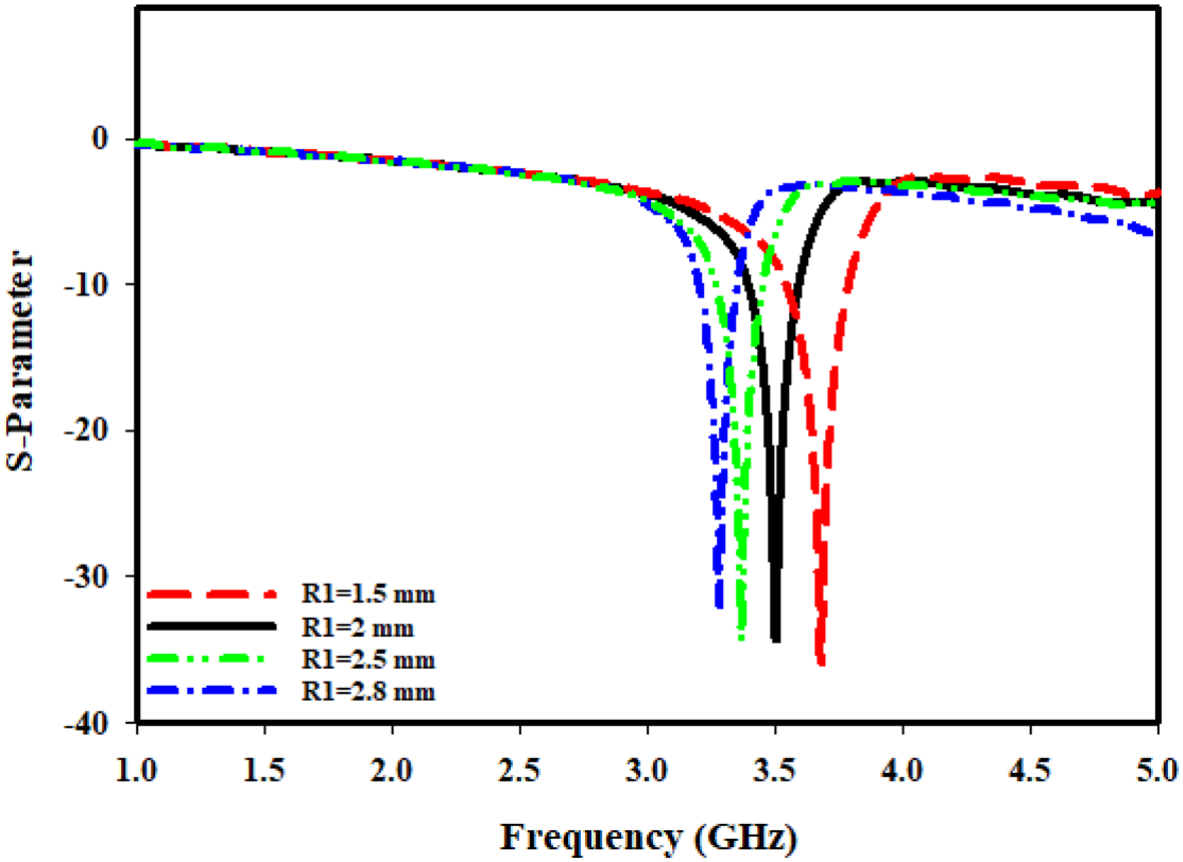
Transmission coefficients tuning when repositioning the ports in the X-direction for L4.

**Figure 12:**
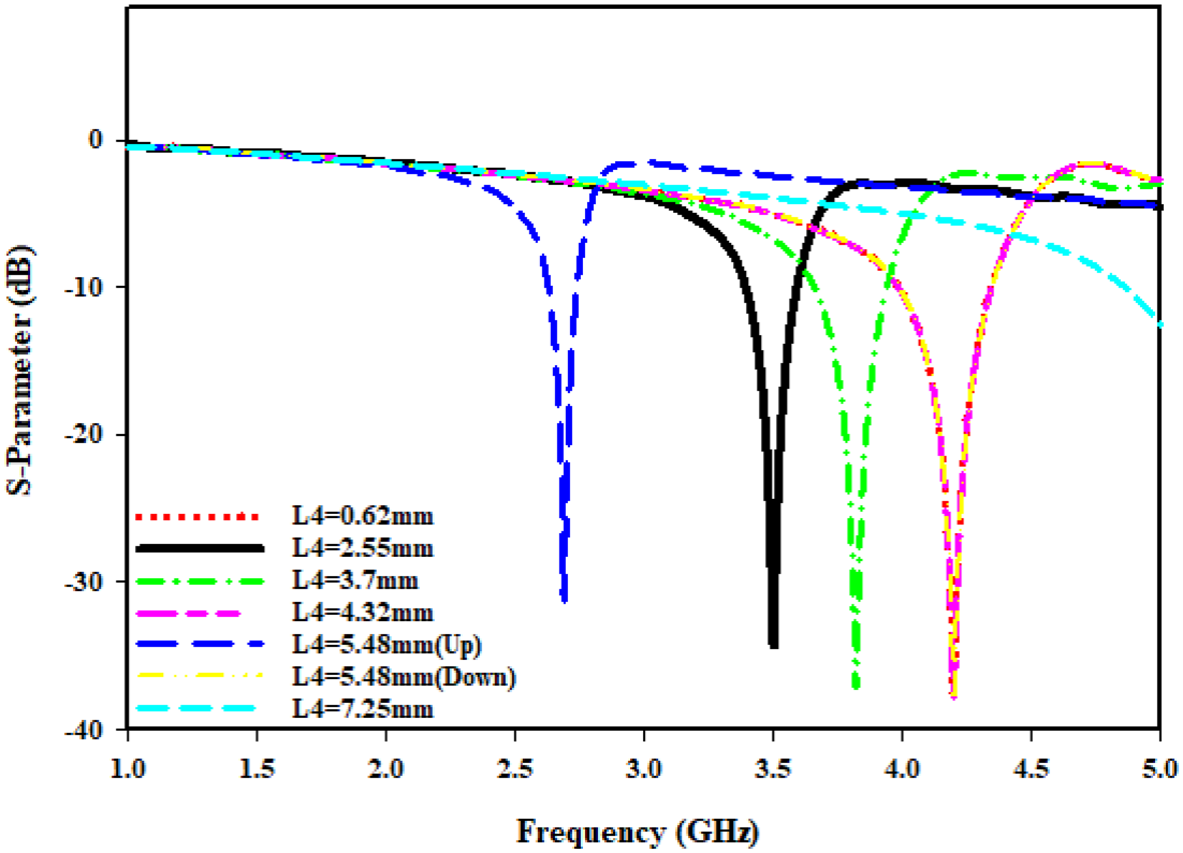
Transmission coefficients tuning when repositioning the ports in the X-direction for R1.

In cases where the findings are obtained earlier, the port is orientated along Z-axis. While shifting ports in X-direction and Y-direction, it is vital to look into how different port placements affect tuning characteristics, scattering, and effective parameters. The ports are arranged in X-direction, as well as the magnetic and electric fields have been aligned in Y-direction and Z-direction, respectively, as Fig. 10b illustrates. The transmission coefficient (S21) can be seen from Fig. 11, where the slotted-strip line length is varied. At the center frequency of 4.993 GHz, reflection coefficient is − 12.532 dB in the case where slotted-strip line length has a L4 = 7.25 mm value. In contrast, the slotted material displays resonances at 4.187 GHz with transmission coefficients of − 36.696 dB when its length is reduced to 0.62 mm. In the case when slotted-strip line length is lowered to L4 = 2.55, 3.7, and 4.32 mm, the line exhibits resonance frequencies of 3.51, 3.8, and 4.194 GHz with a reflection coefficient of − 34.512, − 37.326, and − 37.844 dB, respectively. The slotted-strip line length shows reflection coefficients regarding − 31.488 and − 37.634 dB at the 2.687 and 4.208 GHz center frequencies in the case when it is shortened to L4 = 5.48 mm from both up and down the strip line. As the radius from the center to inner ring is changed, Fig. 12 displays the transmission coefficient. The coefficient of reflection at center frequency of 3.271 GHz is − 31.88 dB when the radius of MTM to the inner ring has a value of R1 = 2.8 mm. In contrast, resonances at 3.681 GHz with transmission coefficients of − 35.884 dB are seen if the radius is reduced to 1.5 mm. It shows that the reflection coefficients at the 3.509 and 3.368 GHz center frequencies are − 34.428 and − 34.302 dB, respectively, in the case when the radius is lowered to R1 = 2 and 2.5 mm. Frequency and band-width impacts of adjusting slotted split ring while the wave moves down X-axis are summarized in Tables 4 and 5.

**Table 4:** Performance comparison of the proposed MTM for different slotted-strip line lengths (L4), and Radius from the center (R1) in Z-direction.

L4 (mm)	Frequency (GHz)	Bandwidth (GHz)	R1 (mm)	Frequency (GHz)	Bandwidth (GHz)
0.62	4.14	3.824–4.272	1.5	3.592	3.385–3.677
2.55	3.5	3.3–3.52	2	3.5	3.3–3.52
3.7	3.714	3.494–3.8	2.5	3.209	3.094–3.266
4.32	4.14	3.82–4.28	2.8	3.058	2.969–3.094
5.48 (up)	2.637	2.566–2.69			
5.48 (down)	4.155	3.824–4.274			
7.25	4.994	4.762–5.0

**Table 5:** Performance comparison of MTM for different slotted-strip line lengths and the radius in the X-direction.

L4 (mm)	Frequency (GHz)	Bandwidth (GHz)	Resonance Peak (dB)	R1 (mm)	Frequency (GHz)	Bandwidth (GHz)	Resonance Peak (dB)
0.62	4.187	3.979–4.354	− 36.696	1.5	3.681	3.542–3.792	− 35.884
2.55	3.51	3.382–3.604	− 34.512	2	3.509	3.37–3.52	− 34.428
3.7	3.80	3.659–3.944	− 37.326	2.5	3.368	3.264–3.451	− 34.302
4.32	4.194	3.986–4.347	− 37.844	2.8	3.271	3.194–3.354	− 31.88
5.48 (up)	2.687	2.624–2.743	− 31.488				
5.48 (down)	4.208	3.98–4.353	− 37.634				
7.25	4.993	4.854–5.0	− 12.532				

## Analysis, and discussion the results

This portion employs the CST microwave studio post-processing module for the extraction of effective parameters regarding suggested MTM unit cell. It does this by using the robust retrieval approach and knowledge of S21 and S11^32,33^. After then, the data is examined. For various resonances, the features of electric field, surface current, and magnetic field are looked at. The suggested MTM is contrasted with some of the current initiatives in this section.

### Analyses of electric and magnetic fields, and surface current

**Figure 13:**
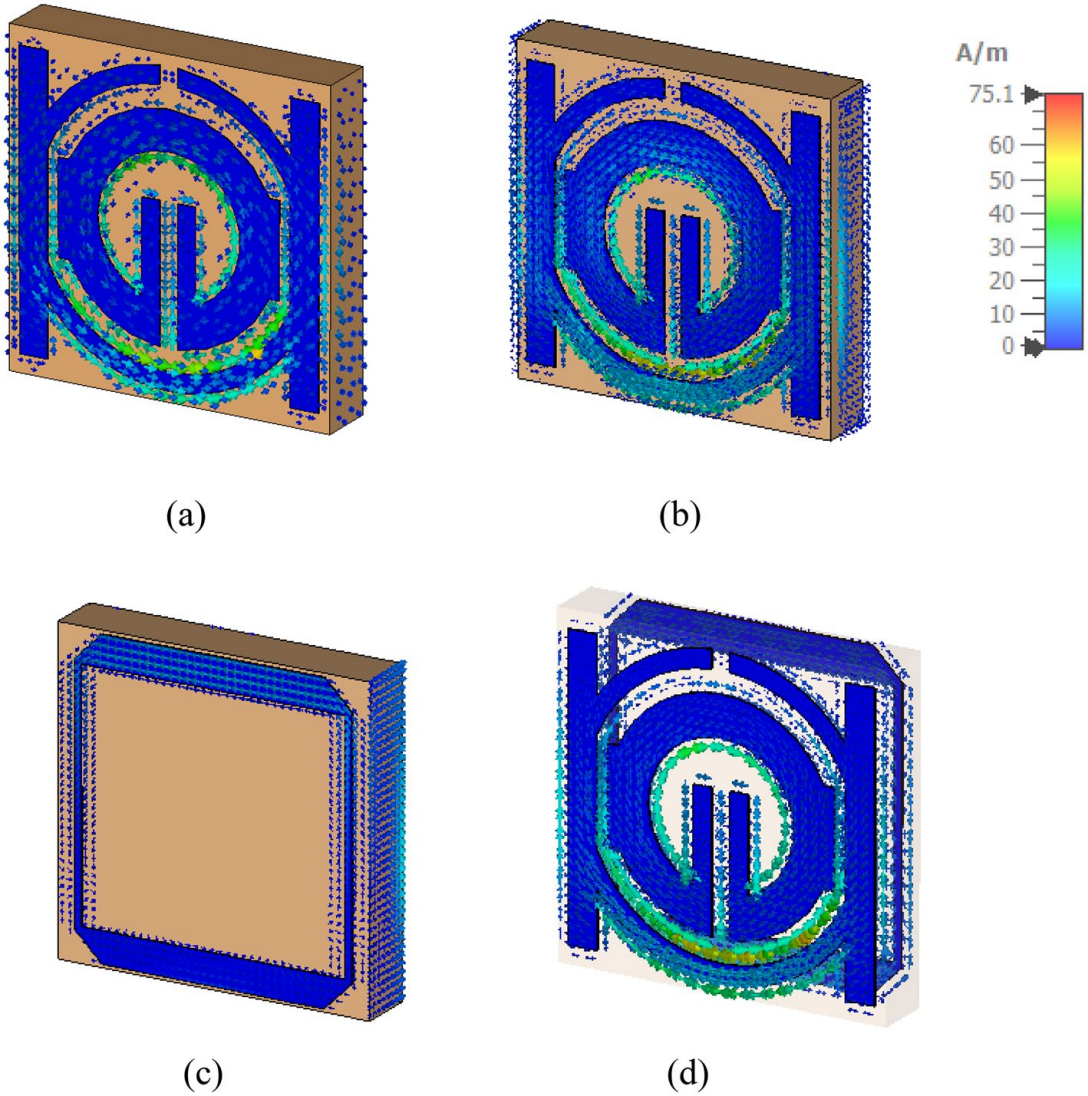
Analysis current distribution for proposed-MTM; (**a**) SNG-MTM; (**b**) front view; (**c**) back view for DNG-MTM; and (**d**) front and back view for copper of DNG-MTM for resonant frequency 3.5 GHz.

**Figure 14:**
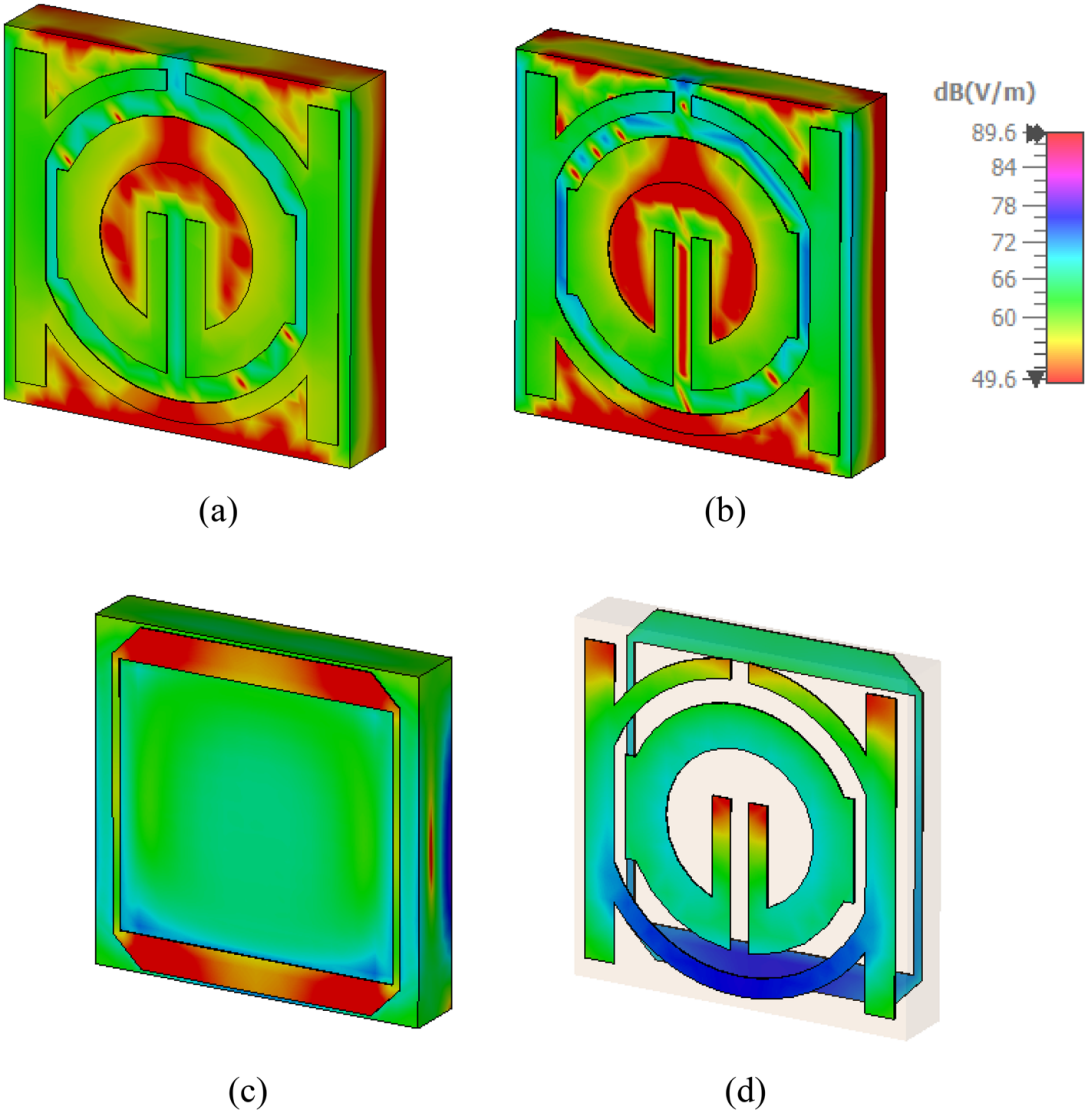
Analysis the magnetic field distribution for proposed-MTM; (**a**) SNG -MTM; (**b**) front view; (**c**) back view for DNG-MTM; and (**d**) front and back view for copper of DNG-MTM for resonant frequency 3.5 GHz.

**Figure 15:**
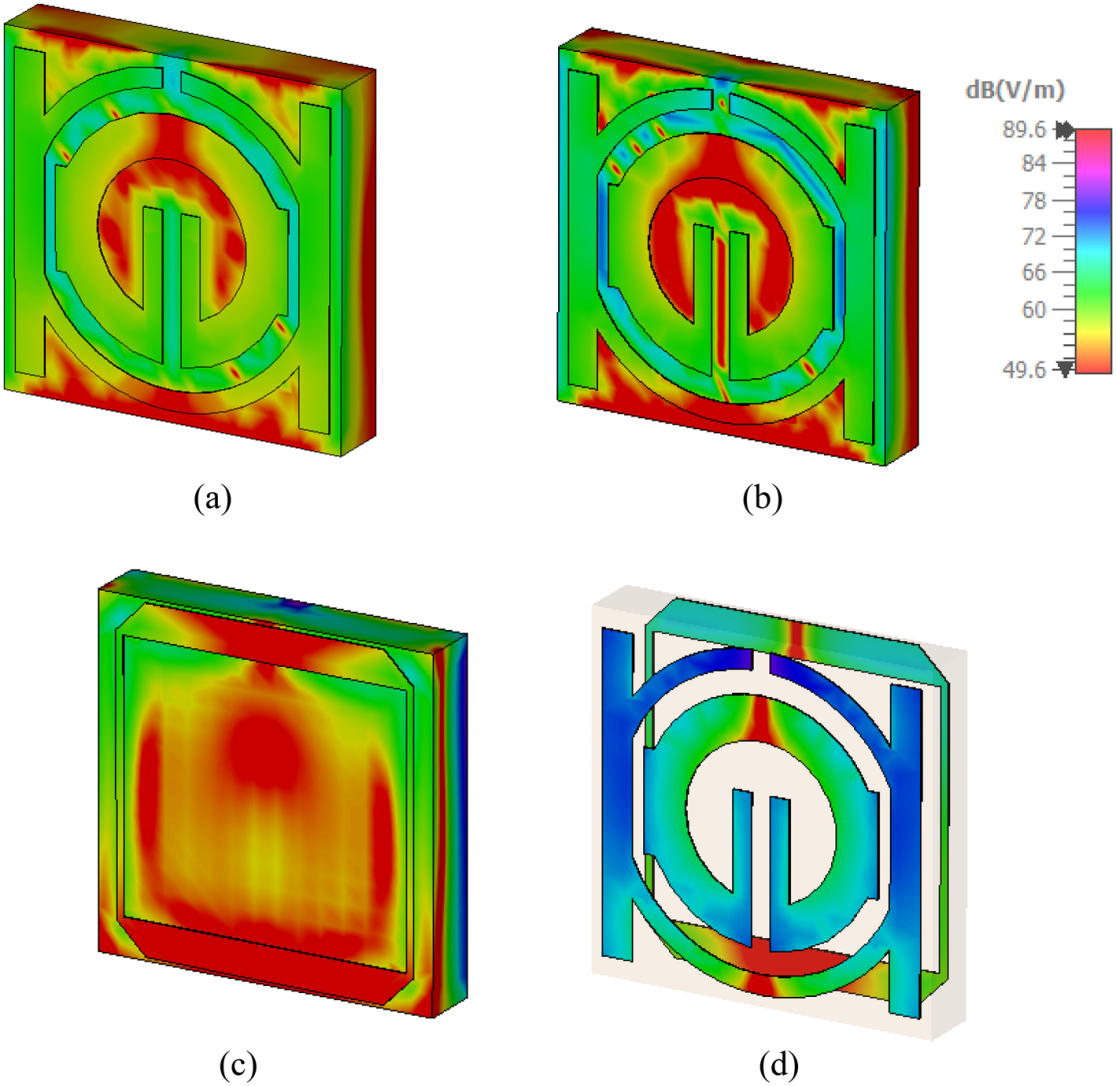
Analysis the electric field distribution for proposed-MTM; (**a**) without slotted square BG; (**b**) front view; (**c**) back view; and (**d**) front and back view for DNG-MTM with slotted-square BG for resonant frequency 3.5 GHz.

Research on magnetic, electric, and surface current fields could help elucidate a metamaterial's characteristics. An incident wave at great distance from identified MTM component can be modeled by applying plain wave with the linear excitation through two waveguide ports. A Gaussian pulse is used as the input signal, and open boundary condition has been provided in direction of the incidence. The commonly recognized hypothesis states that, regardless of geometrical design, electromagnetic resonance is created by an LC equivalent circuit^34^. In addition, induced currents show the inductance L, whereas a division or gap indicates capacitance C. As seen in Fig. 13, the current regarding the suggested metamaterial concentrates around splitter rings and on right and left sides in the front view of the structure and the slot of tuning that results from capacitive effect activation. In addition to all that, it has been evident that the focus of the current is on the square slotted patch in the design's back aspect^35^. Lowing currents create the magnetic field that surrounds these areas, as shown in Fig. 14. Strong electric fields have been observed around the resonator and back slotted square patch in back view at this frequency, which has been shown in Fig. 15.

### Analyses of the effective parameters

Figures below illustrate transmission as well as coefficients of reflection, permeability, permittivity, and normalized impedance of suggested metamaterial unit cell for negative MTM for both types (DNG and SNG), in which and magnetic and electric fields have been aligned in x-axis and y-axis, respectively, and signal travels on Z-axis. The equations used to calculate the effective parameters are given as below^36,37^;

5 $${S}_{11}= \frac{{R}_{01} \left(1- {e}^{j2n{k}_{o}d}\right)}{\left(1- {R}_{01}^{2} {e}^{j2n{k}_{o}d}\right)}$$

6 $${S}_{21}= \frac{ \left(1- {e}^{j2n{k}_{o}d}\right)}{\left(1- {R}_{01}^{2} {e}^{j2n{k}_{o}d}\right)}$$

where *S11* and *S21* are the S-parameters for reflection and transmission through the structure respectively, *m* is an integer and *k*_*o*_ is the wave vector in free space, which ac- counts for the different possible branches of the inverse cosine function.

7 $${R}_{01}= \frac{\left(Z-1\right)}{\left(Z+1\right)}$$

8 $$Z= \pm \sqrt{\frac{{\left(1+ {S}_{11}\right)}^{2}- {S}_{21}^{2} }{{\left(1- {S}_{11}\right)}^{2}- {S}_{21}^{2}}}$$

9 $$n= \frac{1}{{k}_{o} d} \left\{\left[Im. \left[ln{e}^{j2n{k}_{o}d}\right]+2\pi m\right]-j[Re.\left[ln{e}^{j2n{k}_{o}d}\right]]\right\}$$

10 $$\varepsilon = \frac{n}{z}$$

11 $$\mu =nZ$$

where *Im*.(.) represents imaginary part, *Re*.(.) real part, *ε* is the permittivity, *μ* the permeability, *d* the thickness of the substrate, *Z* the impedance, and *n* is the refractive index.

**Figure 16:**
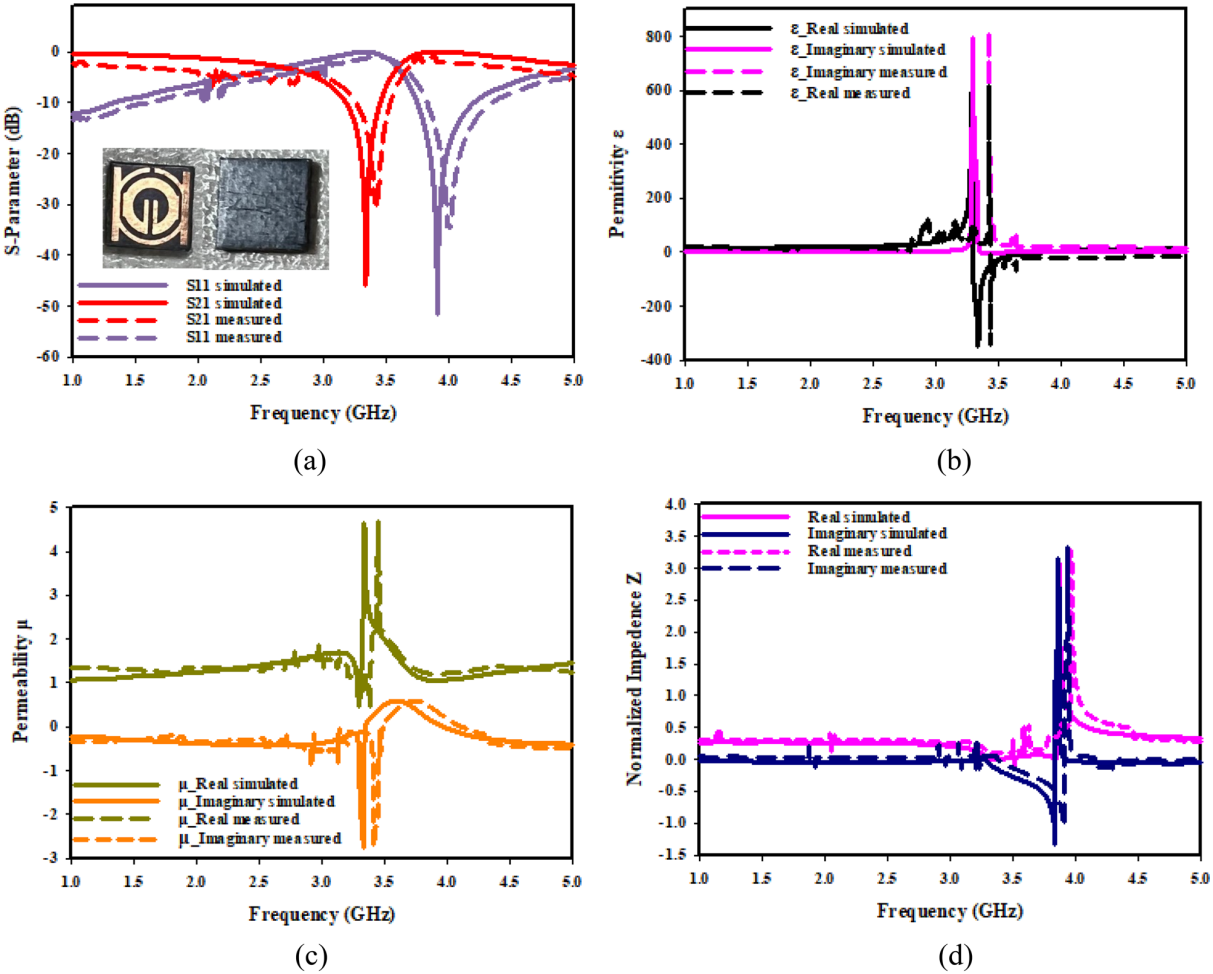
SNG–MTM parameters (**a**) S-parameter; (**b**) permittivity; (**c**) permeability; (**d**) normalized impedance.

The effective parameters regarding a negative near zero index of SNG-MTM and compact SNG-MTM are displayed in Fig. 16. Figure 16a illustrates how electrical resonance is demonstrated by the resonance of S11 after that of S21. The permeability plots in Fig. 16b are almost nil in the relevant band, but the permittivity plots show resonances at 3.5 GHz. The permeability of a material decreases toward zero as a wave's frequency gets closer to its frequency of resonance. The plot of permeability in Fig. 16c shows that 0.44 is the lowest value of permeability. As it has been demonstrated in Fig. 16d, the imaginary and real normalized impedance components are positive in the region of negative permittivity, indicating that suggested MTM operates as passive medium in such ranges of frequency.

**Figure 17:**
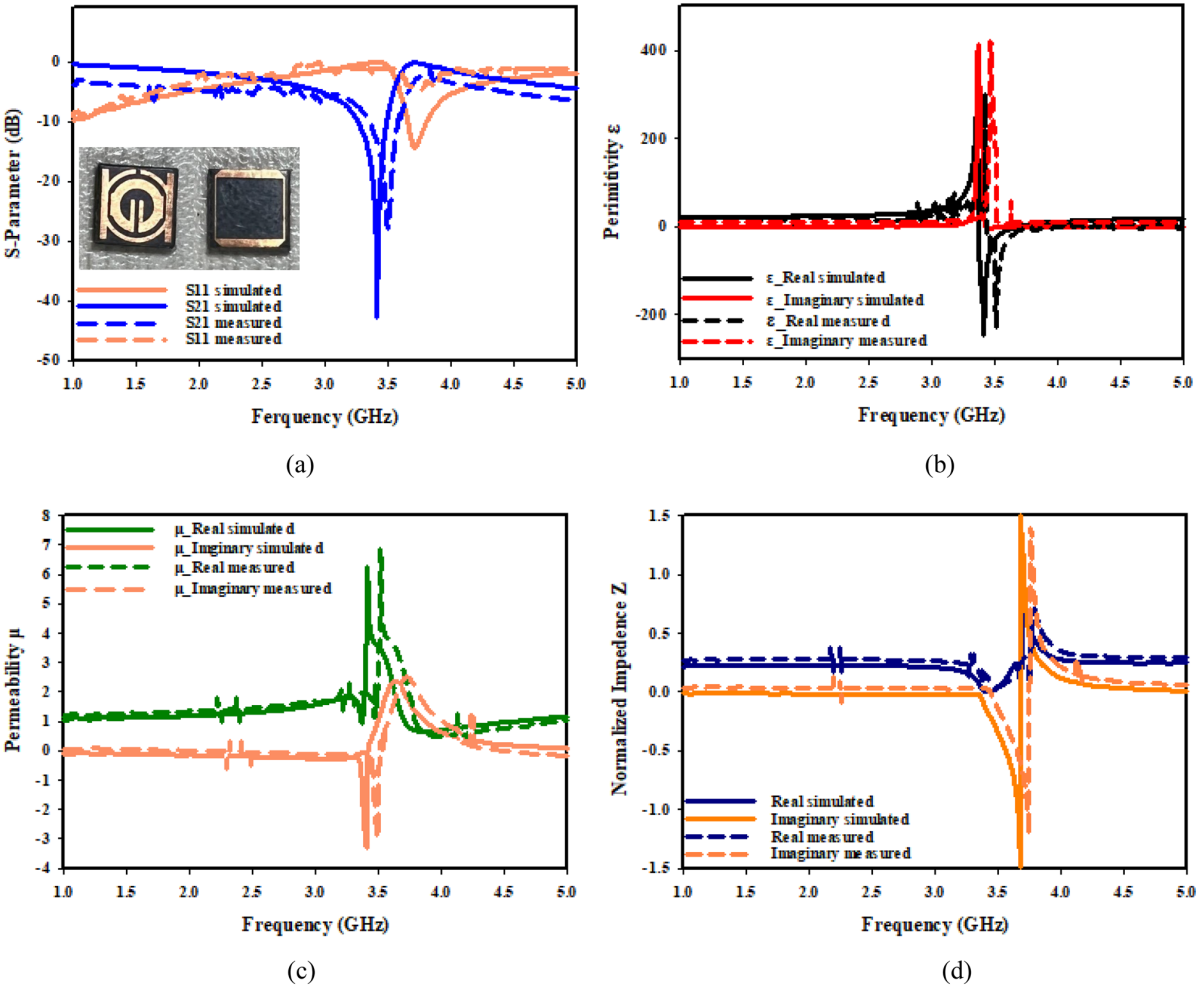
DNG-MTM parameters (**a**) S-parameter; (**b**) permittivity; (**c**) permeability; (**d**) normalized impedance.

The effective characteristics of a double-negative near zero index of MTM are displayed in Fig. 17. Electrical resonance is indicated by the fact that S11's resonance follows S21's, as can be seen from Fig. 17a. The permeability plots in Fig. 17b are almost nil in the relevant band, but the permittivity plots show resonances at 3.5 GHz. A material's permeability diminishes to zero as wave frequency gets closer to resonance frequency. A permeability plot is shown in Fig. 17c, where lowest permeability values are 0.62 and 0.86. Figure 17d illustrates that the suggested functions of MTM as a passive medium in negative permittivity region, as imaginary and real normalized impedance components are positive.

**Figure 18:**
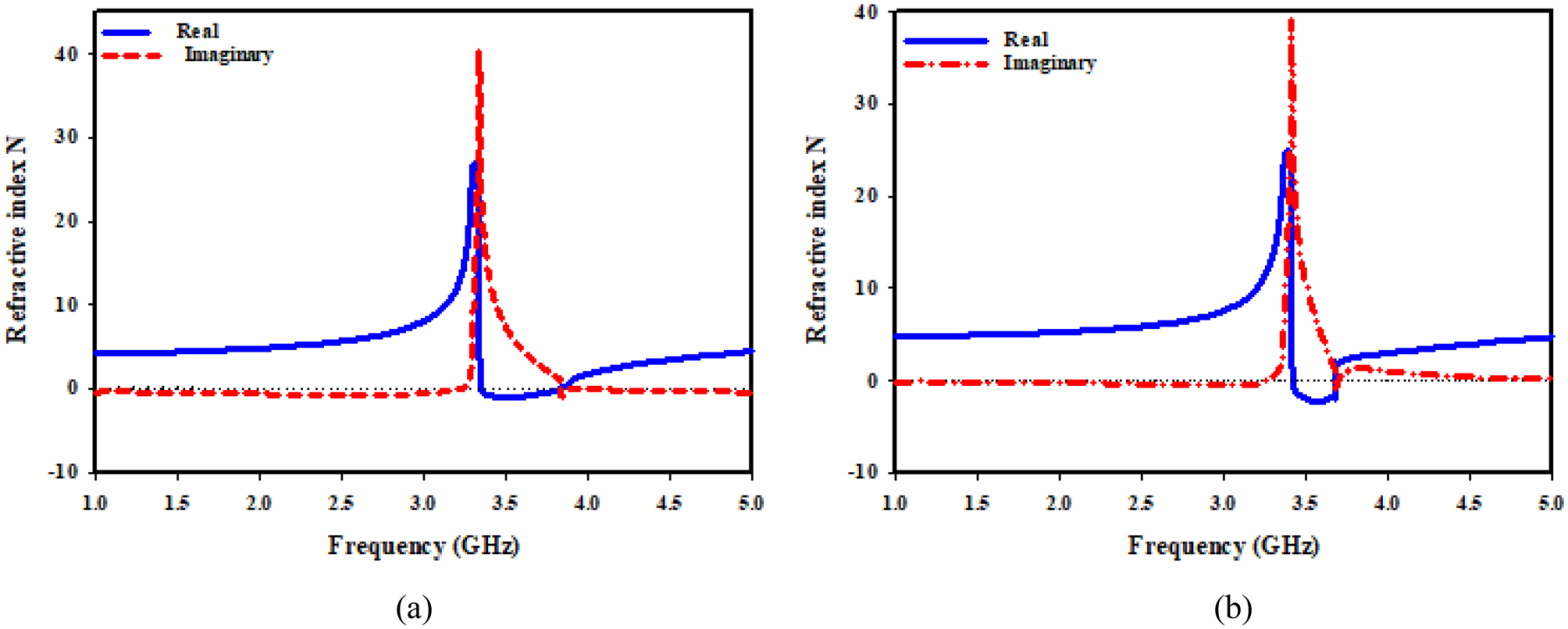
Refractive index of the proposed MTM: (**a**) SNG-MTM; (**b**) DNG-MTM.

The imaginary and real refractive index of suggested unit cell structure for both kinds of MTM is depicted in Fig. 18. As it has been depicted in Fig. 18a, a negative refractive index develops in the range of 3.35–3.85 GHz close to zero. Negative permittivity and negative permeability are prerequisites for materials with a negative value of the refractive index. The result of double-negative (DNG) attribute is the negative refractive index. At the resonant frequency, Fig. 18b shows a transition from positive to negative refractive index. Negative refractive indices are measured in frequency range of 3.41 to 3.68 GHz.

**Figure 19:**
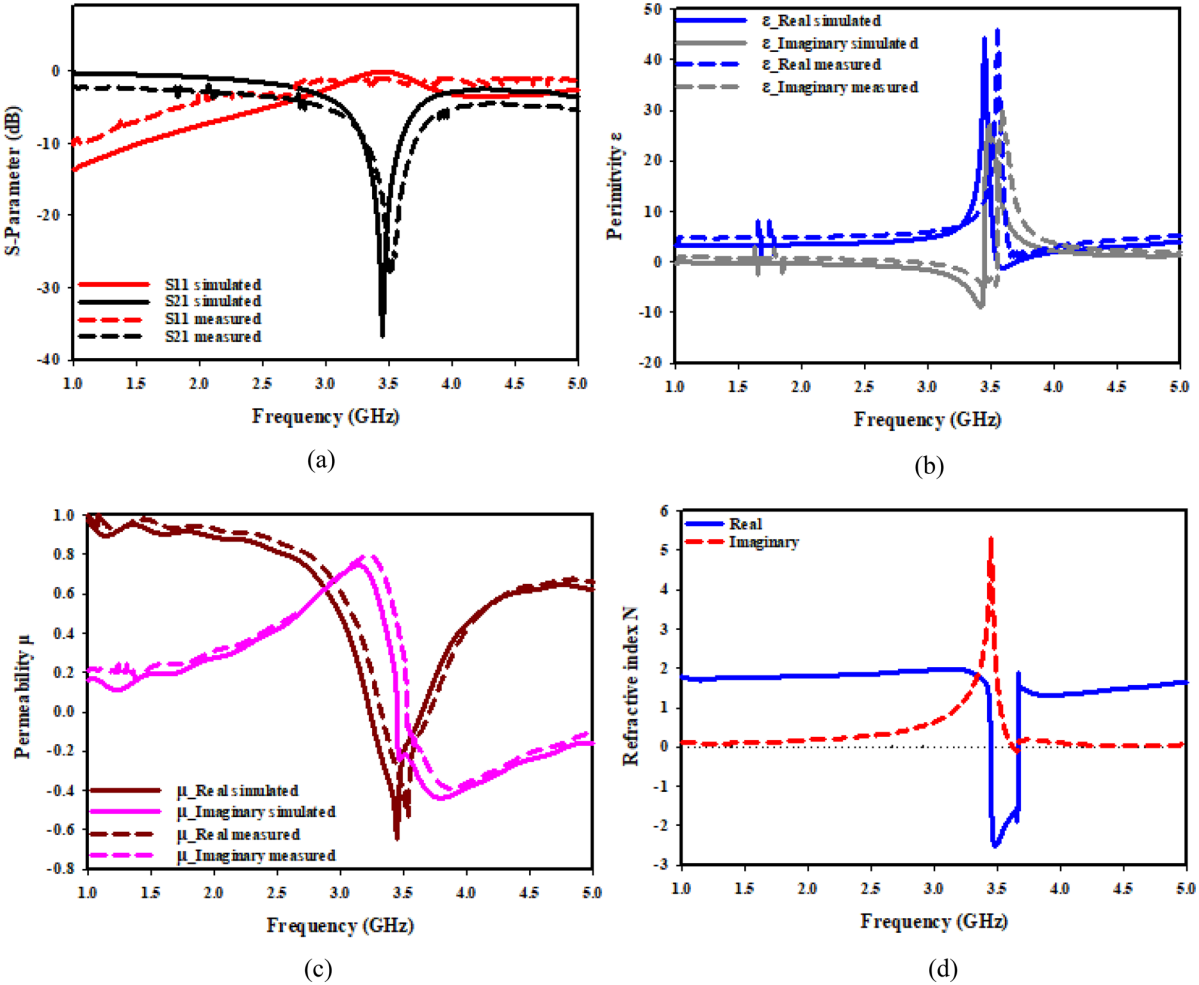
X-axis effective parameter of SNG –MTM: (**a**) S-parameter; (**b**) permittivity; (**c**) permeability; (**d**) refractive index.

**Figure 20:**
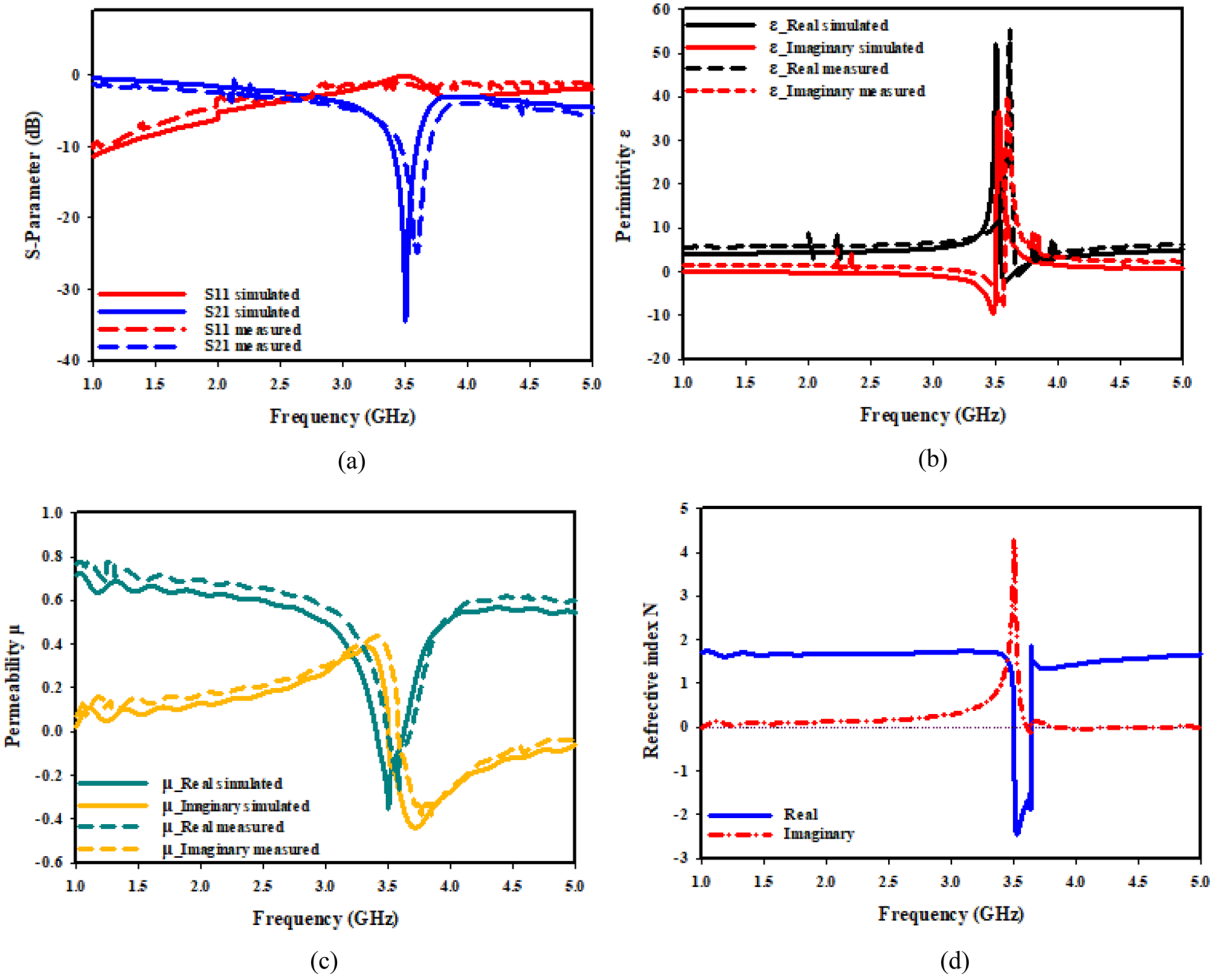
X-axis effective parameter of DNG–MTM: (**a**) S-parameter; (**b**) permittivity; (**c**) permeability; (**d**) refractive index.

Figure 19 displays SNG-MTM design's transmission and reflection coefficients as well as its permeability, permittivity, and refractive index when oriented along the X-axis. As can be shown in Fig. 19a, the metamaterial exhibits a resonant frequency of 3.5 GHz and a peak resonance of − 37.032 dB in the 3.28 − 3.605 GHz region. As permeability starts to decline, Fig. 19b indicates that the real part of permittivity reduces toward zero at 3.5 GHz, whereas Fig. 19c illustrates that permeability reaches a negative value in 3.226–3.654 GHz frequency range. The refractive index in Fig. 19d displays negative values of as low as − 3 for frequencies in the 3.442–3.654 GHz range. Figure 20 displays the permeability, permittivity, coefficients of reflection transmission, and refractive index of DNG-MTM design when it is oriented along X-axis. As can be observed from Fig. 20a, the metamaterial exhibits a resonant frequency regarding 3.5 GHz and a peak resonance of − 34.456 dB in the region of 3.378–3.588 GHz. Figure 20b illustrates how the real part of permittivity approaches zero at 3.5 GHz, with a peak resonance of − 2.72 dB. Meanwhile, Fig. 20c demonstrates how permeability reaches a negative value in 3.39–3.64 GHz range of frequency, and permittivity starts to turn positive as permeability starts to decrease. The refractive index in Fig. 20d displays negative values up to − 3 for frequencies in the 3.491–3.641 GHz range.

### Array structure analyses

**Figure 21:**
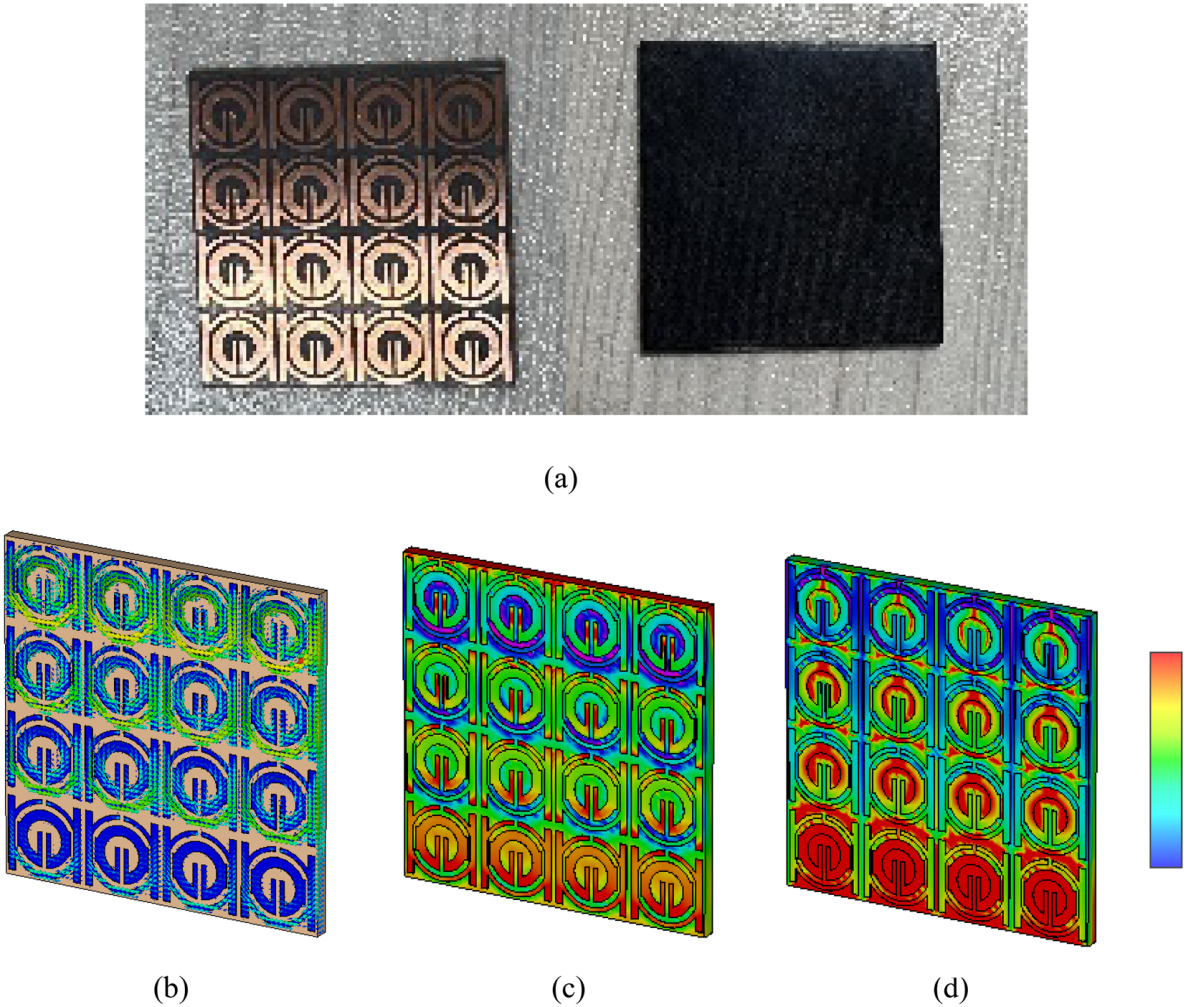
Analysis for (4 × 4) SNG-MTM array: (**a**) fabricated design ofv16 array elements; (**b**) current distribution; (**c**) magnetic field distribution; (**d**) electric field distribution for resonant frequency 3.5 GHz.

**Figure 22:**
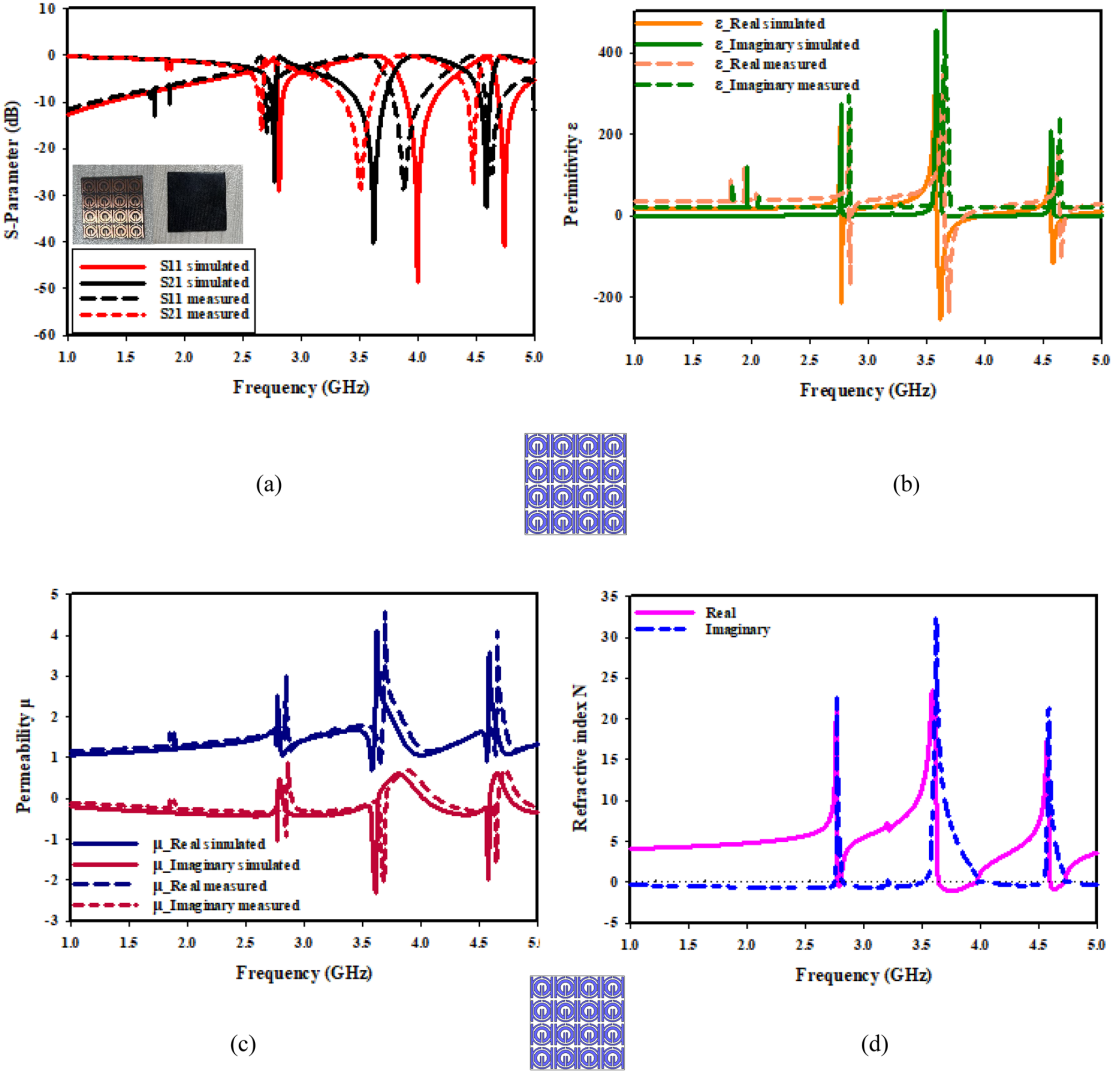
Effective parameters of the (4 × 4) SNG-MTM array structure: (**a**) scattering parameters; (**b**) permittivity; (**c**) permeability; (**d**) refractive index.

This section looks at and discusses the performance of the structure of the unit cell array for SNG-MTM. Effective parameters of the (4 × 4) array structure is displayed in Fig. 21. The fabricated design of 16 array elements is shown in Fig. 21a. Research on surface current, electric, and magnetic fields will help to actualize the metamaterial's properties. Almost all current in the suggested (4 × 4) array structure is concentrated in the bottom cells towards top cells as shown in Fig. 21b, where it flows in the lower and upper sides of outer frame and in the middle of the rings toward slotted-strip line. Unlike the bottom cells, shows that most of the current is dispersed on upper side of outer ring, and the magnetic field around these locations is produced by currents moving in a manner similar to what is shown in Fig. 21c. The distribution of the electric field that has been depicted in Fig. 21d shows that each cell's bottom toward top, and inner splitting ring all experience strong electric fields, which all affect the transmission coefficients regarding capacitance and the variation of the 3.5 GHz resonant frequency. The transmission coefficients of the array are displayed in Fig. 22. With a frequency range of 2.731 to 2.79 GHz and resonant frequencies of 2.763 GHz and transmission coefficients of − 27.589 dB, the (4 × 4) structure in Fig. 22a has two resonant frequencies: 3.597 GHz and 3.449 to 3.718 GHz with a transmission coefficient of − 40.365 dB. Furthermore, there is another resonance frequency at 4.583 GHz, which spans from 4.519 to 4.622 GHz and has a transmission coefficient of − 32.637 dB. Figure 22b displays the imaginary and real effective permittivity of 4 × 4 structure. Permittivity has negative frequency ranges at ranges of 2.756–2.827 GHz, 3.598–3.934 GHz, and 4.559–4.732 GHz, while permeability exhibits values that are almost zero. The (4 × 4) structure possesses both imaginary and real permeability, as shown in Fig. 22c. Nearly zero permeability exists, with three resonance frequencies at 3.5 GHz: 0.642 at 3.574 GHz is the lowest value, while 1.025 at 2.815 GHz is the highest. Figure 22d displays the imaginary and real refractive indices for (4 × 4) array. The ranges of the frequency for the negative value of the refractive index are 2.761–2.81 GHz, 3.624–3.97 GHz, and 4.583–4.732 GHz.

**Figure 23:**
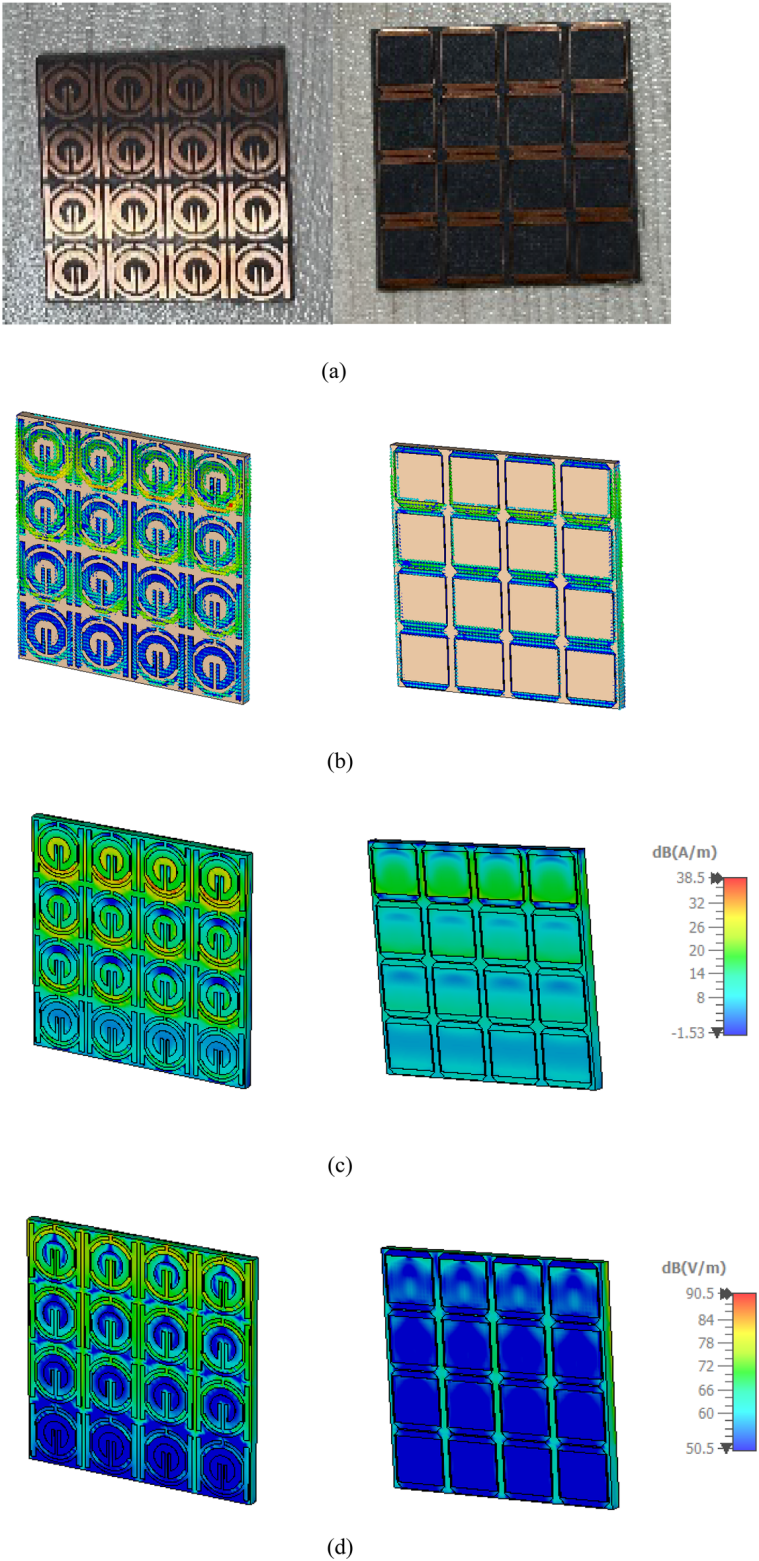
Analysis for (4 × 4) DNG-MTM array: (**a**) fabricated design of 16 array elements (**b**) current distribution; (**c**) magnetic field distribution; (**d**) electric field distribution for resonant frequency 3.5 GHz.

**Figure 24:**
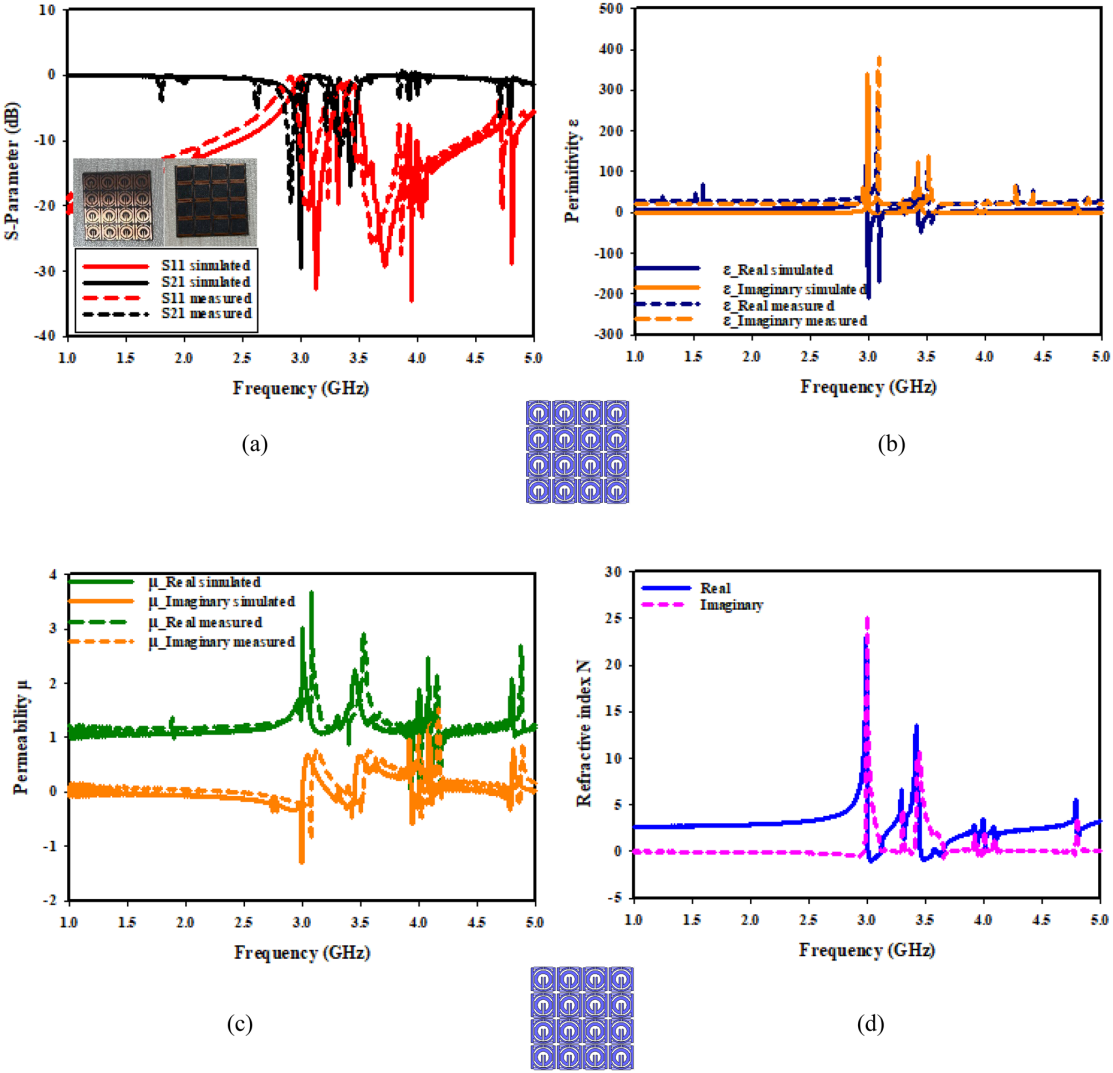
Effective parameters of the (4 × 4) DNG-MTM array structure: (**a**) scattering parameters; (**b**) permittivity; (**c**) permeability; (**d**) refractive index.

The DNG-MTM unit cell array design's performance is evaluated. The effective parameters of (4 × 4) array structure are displayed in Fig. 23. The fabricated design of 16-array elements is shown in Fig. 23a. Research on surface current, electric, and magnetic fields will help to actualize the metamaterial's properties. The proposed (4 × 4) array construction concentrates most of the current in lower toward upper cells, in which it flows strongly in upper and lower sides of outer frame and in center of rings towards slotted-strip line. On the contrary with bottom cells, almost all of the current is distributed on the lower side of outer ring, as it has been depicted in Fig. 23b. The magnetic field surrounding these locations and the slotted square-shaped copper in BG is produced by the movement regarding currents related to that in Fig. 23c. The distribution regarding the electric field in Fig. 23d reveals that there is a notable electric field around the slotted square cooper in BG, at slotted-strip line from top to bottom cells, and on the right and left sides. These factors all contribute to the variation in the transmission coefficients of capacitance and 3.5 GHz resonant frequency. The transmission coefficients of the array are displayed in Fig. 24. As shown in Fig. 24a, the (4 × 4) structure's frequency ranges from 2.954 to 3.023 GHz, with resonant frequencies of 2.991 GHz and transmission coefficients of − 29.80 dB. The second resonant frequency was at 3.46 GHz, with a − 17.243 dB transmission coefficient, and it ranges between 3.389 and 3.472 GHz. Figure 24b displays the imaginary and real effective permittivity of 4 × 4 structure. Permittivity has negative frequency ranges for frequencies of 2.988–3.174 GHz, 3.299–3.326 GHz, and 3.424–3.87 GHz, while permeability exhibits values that are almost zero. The (4 × 4) structure possesses both imaginary and real permeability, as shown in Fig. 24c. Nearly negligible permeability exists, with three resonance frequencies at 3.5 GHz, 0.049 at 4.094 GHz as the lowest value, and 0.011 at 3.932 GHz as the highest. Figure 24d displays the imaginary and real refractive indices for (4 × 4) array. The ranges of frequency for the negative refractive index are 2.987–3.132 GHz, 3.438–3.567 GHz, and 3.596–3.654 GHz.

Table 6 illustrates how the suggested MTM is compared and summarized with some lately published state-of-the-art articles. The most important parameters are thought to be resonant frequency, size, bandwidth, metallic layer, dielectric substrate, ability for being tuned, and material properties like DNG or ENG.

**Table 6:** Proposed MTM comparison with the reported state of art.

Ref	Size (mm^2^)	Freq (GHz)	BW (GHz)	No. of Axes	ENG BW (GHz)	Near- Zero (Mu)	DNG BW (GHz)	Tunable
^38^	10 × 10	3.6	2.5–4.3	1	–	–	Yes 3.74–4.39	No
^39^	10 × 10	3.58, 5.87	–	1	2.988–3.222 3.636–3.996 4.002–4.08 5.898 to 6.0	–	No	No
^8^	8 × 8	2.4, 5.6	2.3–2.49, 5.27–5.87	1	2.42–2.71 5.64–6.15	–	No	No
^40^	8 × 8	3.37, 5.8	3.1–3.6 5.76–5.8	1	3.37–4.5 5.8–5.95	0.02 0.13	No	No
^41^	9.5 × 9.5	2.5, 4.9, 6	2.47–2.52 4.82–4.97 5.9–6.11	1	2–4.03 4.925–4.99 5.955–6.13	–	No	No
**This Work**	9 × 9	3.5	3.30˗3.52	2	yes 3.5–3.718	0.44/(0.62&0.86)	Yes 3.5–3.749	Yes

## Conclusions

This work offers a compact and controllable metamaterial to be used in 5G applications without and with slotted square-shaped copper in Background (DNG and SNG). The metamaterial is composed of metallic circular-shaped split-ring resonator with tunable gap in the center. The suggested metamaterial has a range of operational frequency of 3.30 ~ 3.52 GHz with resonance at 3.5 GHz. Its overall dimensions are 9 × 9 mm^2^. The symmetric structure of the MTM reduces mutual coupling between the array members, and for Z and X primary axis wave propagation, the array exhibits equivalent coefficient of transmission (S21) response to unit cell. Additionally, the suggested MTM's equivalent circuit is modeled in the ADS and validated by using a comparison between CST and the nearly related S21 response. The double-negative index, negative permittivity, and nearly 0 permeability of the MTM features were examined. The contribution of several MTM unit cell components to the resonance has been examined by using surface current, electric field analysis, and magnetic field. The suggested MTM could be used with different wireless devices in 5G sub 6 GHz applications because of its small profile size, negative ENG, high effective DNG, and nearly 0 permeability features. It could be particularly useful for improving the antenna properties. To increase overall performance, next study and design are anticipated to apply the suggested metamaterial to a 5G huge multiple-input multiple-output (MIMO) design of the antenna array to enhance bandwidth, isolation, gain and directivity.

## Data Availability

The datasets used and/or analyzed during the current study available from the corresponding author on reasonable request.
